# Evaluating the usability of a co-designed power assisted exercise graphical user interface for people with stroke

**DOI:** 10.1186/s12984-023-01207-7

**Published:** 2023-07-24

**Authors:** Rachel Young, Karen Sage, David Broom, Andrew Hext, Nicky Snowdon, Christine Smith

**Affiliations:** 1 Department of Allied Health Professions, Advanced Wellbeing Research Centre, Sheffield Hallam University, 2 Old Hall Road, Sheffield, S9 3TU UK; 2 Faculty of Health and Education, Manchester Metropolitan University, Manchester Brooks Building, 53 Bonsall Street, Manchester, M15 6GX UK; 3 Centre for Sport Exercise and Life Sciences, Institute of Health and Well-Being, Coventry University, Coventry, CV1 2DS UK; 4 Sports Engineering Research Group, Advanced Wellbeing Research Centre, Sheffield Hallam University, 2 Old Hall Road, Sheffield, S9 3TU UK; 5 College of Health, Wellbeing and Life Sciences, Sheffield Hallam University, Collegiate Crescent Campus, Sheffield, S10 2BP UK; 6 Advanced Wellbeing Research Centre, Sheffield Hallam University, Collegiate Crescent Campus, Sheffield, S10 2BP UK

**Keywords:** Assistive technology, Co-design, Graphical user interface, Power assisted exercise, Rehabilitation technology, Stroke, Usability evaluation

## Abstract

**Background:**

Digital advancement of power assisted exercise equipment will advance exercise prescription for people with stroke (PwS). This article reports on the remote usability evaluation of a co-designed graphical user interface (GUI) and denotes an example of how video-conference software can increase reach to participants in the testing of rehabilitation technologies. The aim of this study was to evaluate the usability of two sequential versions of the GUI.

**Methods:**

We adopted a mixed methods approach. Ten professional user (PU) (2M/8F) and 10 expert user (EU) participants (2M/8F) were recruited. Data collection included a usability observation, a ‘think aloud’ walk through, task completion, task duration and user satisfaction as indicated by the Post Study System Usability Questionnaire (PSSUQ). Identification of usability issues informed the design of version 2 which included an additional submenu. Descriptive analysis was conducted upon usability issues and number of occurrences detected on both versions of the GUI. Inferential analysis enabled comparison of task duration and PSSUQ data between the PU and EU groups.

**Results:**

Analysis of the ‘think aloud’ walkthrough data enabled identification of 22 usability issues on version 1 from a total of 100 usability occurrences. Task completion for all tasks was 100%. Eight usability issues were directly addressed in the development of version 2. Two recurrent and 24 new usability issues were detected in version 2 with a total of 86 usability occurrences. Paired two tailed T-tests on task duration data indicated a significant decrease amongst the EU group for task 1.1 on version 2 (P = 0.03). The mean PSSUQ scores for version 1 was 1.44 (EU group) and 1.63 (PU group) compared with 1.40 (EU group) and 1.41 (PU group) for version 2.

**Conclusions:**

The usability evaluation enabled identification of usability issues on version 1 of the GUI which were effectively addressed on the iteration of version 2**.** Testing of version 2 identified usability issues within the new submenu. Application of multiple usability evaluation methods was effective in identifying and addressing usability issues in the GUI to improve the experience of PAE for PwS. The use of video-conference software to conduct synchronous, remote usability testing is an effective alternative to face to face testing methods.

**Supplementary Information:**

The online version contains supplementary material available at 10.1186/s12984-023-01207-7.

## Background

### Power assisted exercise

Engagement in physical exercise following stroke is associated with improved mobility, aerobic fitness, muscular strength and psychosocial wellbeing [1–3]. Importantly, aerobic exercise sustained at a moderate to high intensity reduces vascular and metabolic risk factors for recurrent stroke [4]. Guidelines on the optimal intensity, type and duration of exercise for People with Stroke (PwS) have been published [5]. However, people with complex motor impairment resulting from stroke experience difficulties in accessing conventional exercise equipment [6] and motor impairment can impede achievement of the required intensity for health benefits [7]. Whole body power assisted exercise equipment manufactured by Shapemaster© Global Ltd is safe and accessible for people with complex neurological impairments [8] or profound intellectual and multiple disabilities [9]. Shapemaster Global Ltd© operate a global distribution model through which the power assisted exercise equipment is purchased by providers of leisure, community and rehabilitation services. The recommended operating model comprises a circuit of between 8 and 12 machines and users transition around the circuit in sequence. Evaluation of the equipment amongst a sample of older adults indicated improved strength and balance associated with a 12-week programme of power assisted exercise [10]. PwS who engage in power assisted exercise (PAE) report physical and psychosocial benefits [11] and assisted cycling is known to improve aerobic fitness following stroke [12]. PwS and rehabilitation professionals identified that the development of effort detection technology synchronised with the power assisted exercise equipment would enable users to access a tailored exercise prescription and gain real time feedback on their exercise performance [13].

Individualised performance targets with real time feedback to optimise goal attainment has been identified as a priority in the design and development of technologies for PwS [14]. Biofeedback has been synchronised with gaming and virtual reality programmes to enhance the experience and efficacy of stroke rehabilitation interventions [15–18]. The development of assistive technologies in stroke rehabilitation is rapidly evolving; meaningful public involvement in their design, testing and evaluation is essential to ensure implementation of effective products which are fit for purpose in the intended setting [19].

### Medical device technology framework

This study reports on the usability of a high fidelity, prototyped graphical user interface (GUI) designed to provide feedback on exercise performance using effort detection technology on the power assisted exercise equipment. The four-stage medical device technology framework proposed by Shah et al. [20] was adopted to ensure a user-centred, iterative approach towards the co-design and usability evaluation of the new technology. The framework was previously adopted to design and test a novel fall detection system for older adults [21, 22]. In their example, early mock ups were used to stimulate discussion during focus groups with representative users during stages one and two [22]. The design and testing of a novel flexible functional electrical stimulation system for upper limb functional activity practice was also underpinned by the medical device technology framework [23] and included development of a model to predict set up time for the technology [24]. In the project reported in this article, stages one and two comprised user engagement and co-design methods with regular input from expert user and professional user groups [13]. The outcome of the first two stages was a co-designed GUI which enabled users to select and navigate through a range of power assisted exercise programmes, and view real time feedback on their exercise performance. This article reports on stage three of the process which comprised a two-part procedure to examine the usability of two versions of the GUI.

Alternative design approaches include the double diamond design process model [25] which was adopted to design and test a new interface for ‘Stappy,’ a sensor feedback system for walking performance. Prototype devices were introduced to participants during the initial discovery phase of the design cycle to stimulate discussion focussed upon user requirements [26]. Cultural probes have been introduced in previous user centred design examples to develop stroke technologies intended for use in the home environment [27], however, PAEE is typically used in leisure or rehabilitation venues rather than the individual’s setting. The Medical Device Technology Framework [20] emphasises inclusion of multiple end user groups comprising expert users (EU) who live with health changes and professional users (PU) defined as the professionals involved in the implementation and prescription of the technology. Commercialisation and continued development of new technologies is directly considered in stage 4.

### Usability evaluation

Assistive technologies can enable PwS to independently perform functional activities and rehabilitation technologies are designed to facilitate achievement of therapeutic goals [28]. Ease of use has been identified as a strong predictor of intention to use a particular technology [29]. Usability evaluation of new rehabilitation technologies enables identification of recurrent usability issues, measurement of task duration and evaluation of user satisfaction [30]. It calls for representative users to perform representative tasks to identify the strengths and shortfalls of a device in order to bring about improvements [31]. Technologies for PwS previously evaluated through usability testing include an assistive game controller [32], sensor feedback system for gait [26], wearable functional electrical stimulation garments [33] and virtual reality gaming system [34]. Data collection methods which have been implemented in the testing of novel assistive technologies include user satisfaction questionnaires [35, 36], task completion [25, 26], task duration [25] and comparison between different devices [23]. Recurrent usability issues include difficulty donning and doffing [32, 33], failure to complete tasks [37] and difficulty accessing emergency stop function [26, 33].

The importance of trust in assistive and rehabilitation technologies for PwS has been emphasised and features which facilitate sustained successful engagement include task variety, clear communication, fatigue management and reward [35]. Usability evaluation is central to the development of acceptable and meaningful technologies which will be adopted by service providers and utilised by end users [31]. Usability testing has historically been an in-person activity where participants and researchers co-locate [38]. The Covid-19 pandemic accelerated engagement with communication technologies and the research community has shifted from face-to-face methods of data collection to increased use of video-conferencing software [39]. The study reported in this article represents an example of how remote methods of usability testing can increase reach to users of rehabilitation technologies [38] and represents a potential solution to the challenges associated with recruitment of participants for face-to-face testing methods.

### Overview of article

The study reported in this article recruited representative user groups to evaluate the usability of two sequential versions of the co-designed GUI to optimise the usability and functionality of the new technology. For the purposes of this manuscript, usability is defined as “the effectiveness, efficiency*,* and satisfaction with which specified users achieve specified goals in particular environments” [30]. Users in the context of this study are either PU, i.e. rehabilitation professionals or clinical exercise physiologists, or EU i.e. PwS, including people who have prior experience of PAE equipment. The methods section defines four objectives which underpinned the study and describes the synchronous remote usability testing procedure conducted on two sequential versions of the co-designed GUI. The approaches adopted to collect and analyse quantitative and qualitative data are explained and justified. The results section reports on the findings and is organized according to the four underpinning objectives. The findings and their interpretation are explored in the discussion section and compared with previous relevant examples in the published literature.

## Methods

### Aim

The aim of this study was to evaluate the usability of a co-designed GUI to enable PwS and rehabilitation professionals to effectively utilise power assisted exercise equipment. The objectives were to: (1) evaluate the usability of version 1 of the GUI; (2) use the findings from version 1 to develop and evaluate a second iteration (extended version) of the GUI; (3) compare the usability of version 1 with version 2; (4) Analyse usability as experienced by EU and PU’s.

To achieve this aim, we adopted a mixed methods approach. Quantitative methods were used to examine task completion, task duration and user satisfaction using the Post Study System Usability Questionnaire (PSSUQ) [40]. Task completion is a strong indicator of the usability of digital rehabilitation technologies [41] and task duration data provides an indication of set up time which is a key determinant in the adoption of rehabilitation technologies [24, 42]. The PSSUQ was selected to measure user satisfaction as it distinguishes between system usability, quality of information and quality of the interface [40]. ‘Think aloud’ was adopted as a qualitative method to gain insight into the users’ experience of navigating the GUI and identify specific usability issues [21]. All usability evaluations were conducted with both EU and PU.

**Fig. 1:**
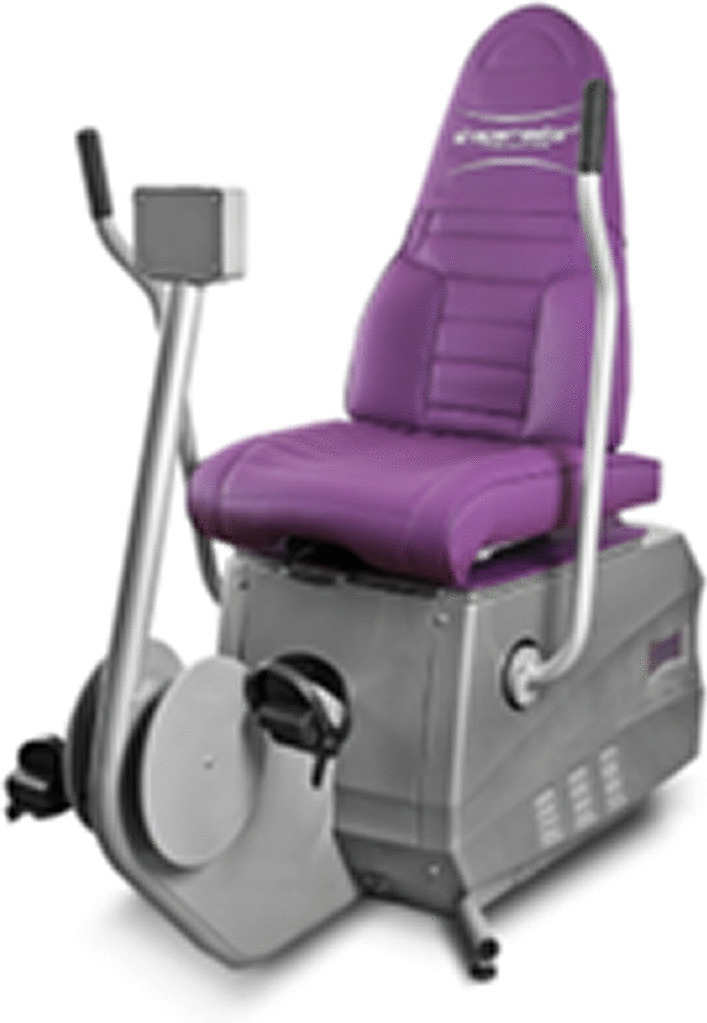
Cross cycle: co-designed GUI was intended for this machine

Version 1 of the GUI was specifically designed for the cross-cycle machine (Fig. 1) as previous user involvement indicated that this machine was the most popular [13].

**Fig. 2:**
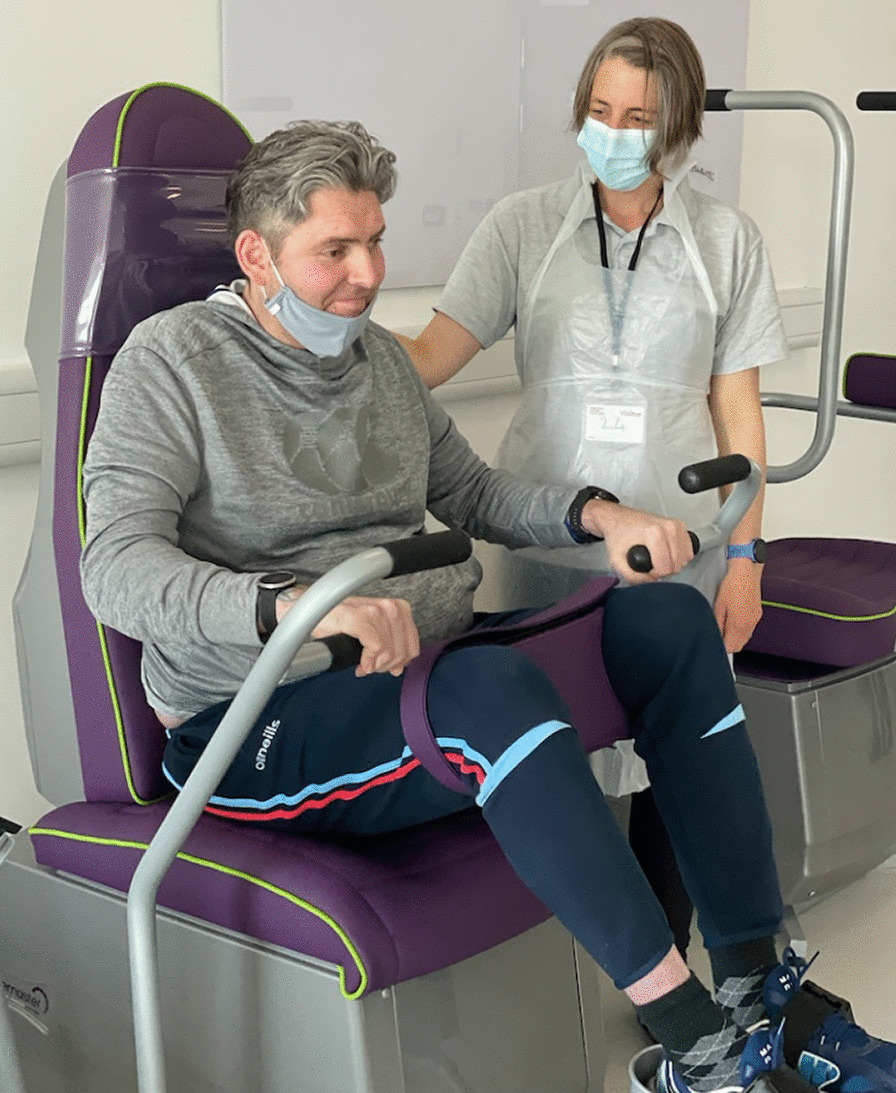
Chest and legs: machine ranked second through consensus methods in use by an EU, supported by PU

It was envisaged that the GUI would be adapted to the range of machines manufactured by Shapemaster Global. Figure 2 is an image of the chest-and-legs machine which was ranked second most popular through consensus methods [13].

**Fig. 3:**
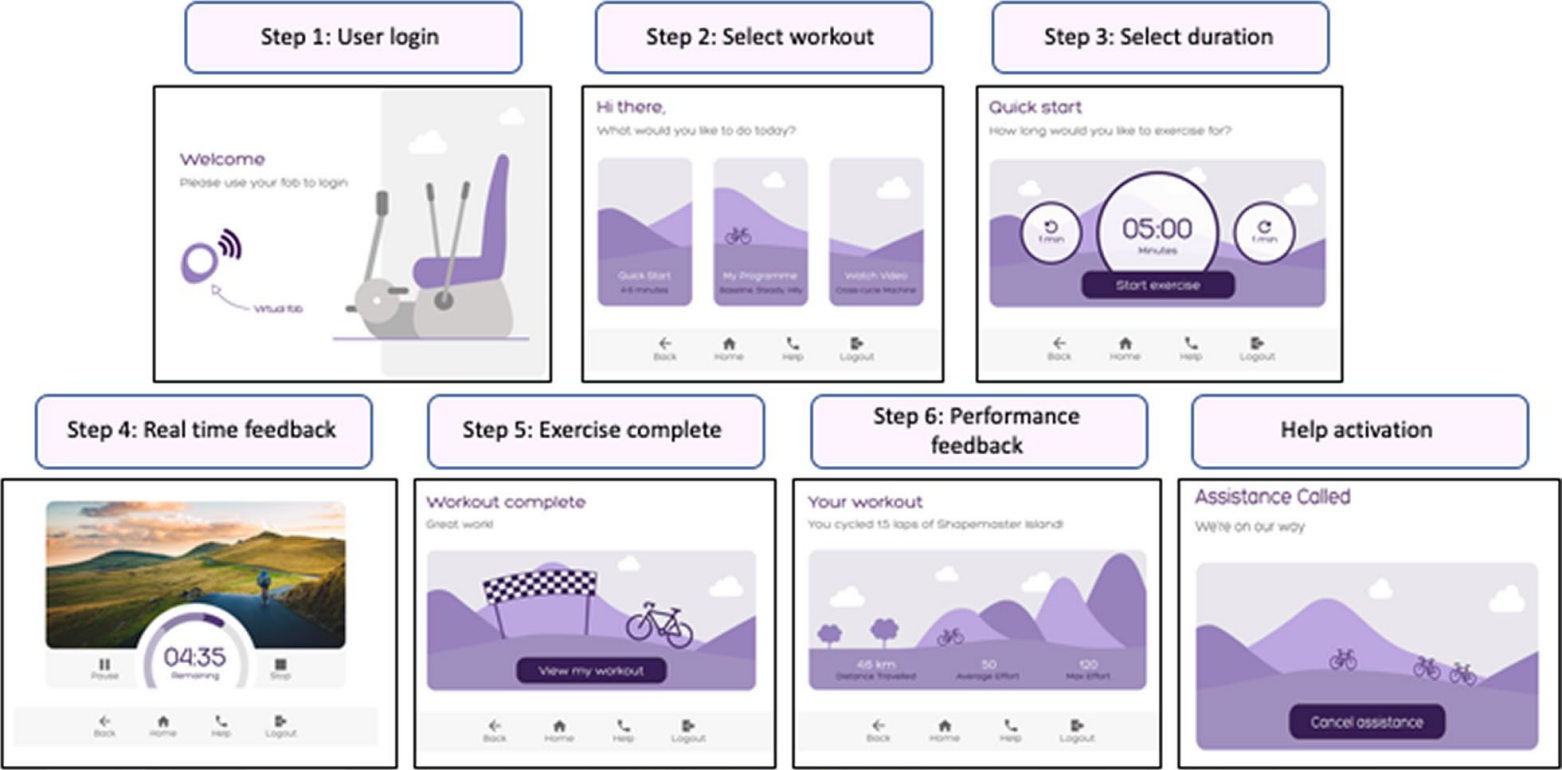
Graphical user interface version 1: this version was created to test the ‘quick start’ programme and help activation function

The version 1 prototype GUI (Fig. 3) comprised 7 sub-menus, namely; (1) user login; (2) programme selection; (3) duration selection; (4) real time feedback; (5) exercise completion; (6) performance feedback; and (7) assistance alert. The real time exercise feedback phase of the programme (step 4) was defaulted to play for a 30 s duration to enable animation of the virtual effort detection display. The virtual effort was displayed on the semi-circular dial with darker shades of purple indicating increased effort. A menu bar at the bottom of the page enabled navigation to the homepage or previous page. This was positioned centrally rather than as a sidebar to account for the spatial awareness impairments which can occur following stroke [43]. Activation of the ‘help’ icon navigated directly to an ‘assistance called’ message intended to assure users that a team member had been alerted.

Both PU and EU’s experiences of using the newly designed GUI were gleaned to identify usability issues to help inform future iterative redesign processes. The usability evaluations consisted of assisted user virtual walkthroughs of the interface using ‘think aloud’ and cursor observation to highlight usability issues, and a post testing user questionnaire, the PSSUQ. Specific tasks were developed to evaluate the usability of the GUI. The tasks were devised to replicate priority functions of the GUI in a real-world setting and incorporated exercise programme selection, programme duration, interpretation of detected user performance and access to assistance. Efficient navigation through the console was deemed a priority to prevent delays or disruption to the operational circuit, therefore timing of task duration was included. Setup time is an important, but neglected research area in the field of rehabilitation technologies [24]. A target of 25 s from user login to exercise commencement was identified as a benchmark by the manufacturer. The use of multiple evaluation tools allowed for the triangulation of data.

Testing was scheduled during a period of government imposed national lockdown in the UK due to the covid-19 pandemic. Virtual versions of the GUI were therefore created in adobe XD and the tests were conducted remotely using Zoom media. Ethical approval for this study was granted by the host university (ER26319972).

The data generated during the first round of usability testing shaped the design of version 2 of the GUI which included extended programme options and comprised 16 submenus.

### Sample size

Preliminary testing has been implemented in usability evaluation to determine the probability of error detection [44]. Due to resource and timescale restrictions this was not feasible and so the probability of error detection was estimated at 0.15. The probabilistic model of problem discovery described by Sauro and Lewis [40] was applied to determine sample size with a target of 95% chance of observation. We therefore aimed for a 95% likelihood of detecting usability problems with an estimated 15% probability of occurrence. A sample size of 19 participants was required [40].

### Recruitment

Convenience sampling was implemented to identify participants for the EU and PU groups. The criteria for participation across both groups was inclusive to capture a range of perspectives and user priorities. The inclusion criteria for the EU representation were; diagnosis of stroke; access to a Wi-Fi connected laptop or digital tablet; able to follow verbal instructions in English; and able to provide informed consent. No prior experience of PAE was stipulated. People who were unable to provide informed consent due to severe cognitive impairment were excluded from participation. Participants for the EU group were identified through a local independent rehabilitation service and the service user network at Sheffield Hallam University. The inclusion criteria for the PU group were; employment relevant to rehabilitation or exercise prescription for people with long term conditions, access to a Wi-Fi connected laptop or digital tablet; able to follow verbal instructions in English and able to provide informed consent. Participants for the PU group were identified through academic teams at the host university, independent practitioners known to the research team and service providers known to the manufacturer.

Potential participants were identified by the lead author (RY) and invited to consider participation via email with an accompanying participant information sheet. The target recruitment was 10 participant per group. Consent was confirmed through completion and submission of an electronic form. Due to the virtual methods of participant recruitment and data collection enforced by the Covid-19 lockdown, detailed assessment of the type and severity of stroke related impairment was not possible.

### Participants

Ten EU participants (6M/4F) and ten PU participants (2M/8F) consented to participate. The mean age of the EU participants was 61.7 years (SD 10.2) and mean time since stroke was 60.9 months (SD 24.7). Fifty percent of the participants had prior experience of PAE and 40% of the participants in the EU group had contributed to prior user involvement and co-design stages of the technology project (Table 1). One participant (EU05) was unable to activate the remote-control mouse icon on Zoom. After several attempts the participant decided to withdraw from the study.

**Table 1:** Expert user participants

Code	Months since stroke	Impairment	Gender	Age (years)	Occupation	Experience of PAE	Earlier co-design participant
EU1	63	Left hemiparesis	Male	62	Medical professor	Yes	No
EU2	76	Ataxia (L > R)	Male	77	Retired farmer	No	No
EU3	73	Right hemi	Male	69	Retired data analyst	Yes	Yes
EU4	86	Right hemi/aphasia	Male	71	Retired sailor	Yes	Yes
EU5	76	Right hemi/aphasia	Female	68	Retired sales manager	Yes	Yes
EU6	9	Left hemi	Female	52	University lecturer	No	No
EU7	24	Quadriparesis	Male	44	Foreman (unable to work since stroke)	No	No
EU8	76	Left hemi	Female	53	Accommodation officer	No	No
EU9	62	Right hemi	Male	65	Engineer	No	No
EU10	64	Left hemi	Female	56	Civil servant	Yes	Yes
Mean months since stroke: 60.9 (SD10.2)			6M/4F	Mean age: 61.7 years (SD 24.7)			

The mean age of participants in the PU group was 42.3 (SD 6.09) years and included representation from sport sciences, rehabilitation physiotherapists and industry. Fifty percent had direct experience of PAE and 60% had contributed to earlier stages of the project (Table 2). A participant in the PU group (PU5) withdrew from the study prior to test two due to work pressures.

**Table 2:** Professional user participants

Code	Occupation	Gender	Age (years)	Experience of PAE	Co-design participant
PU1	Physiotherapist	Female	43	Yes	Yes
PU2	PAE marketing expert	Female	44	Yes	No
PU3	Exercise scientist	Female	36	No	Yes
PU4	Physiotherapist	Female	42	Yes	No
PU5	Occupational Therapist	Female	47	No	No
PU6	Physiotherapist	Female	38	Yes	Yes
PU7	Sport scientist	Male	32	No	No
PU8	Research physiotherapist	Female	45	No	Yes
PU9	Sport scientist	Male	42	No	Yes
PU10	Physiotherapist	Female	54	Yes	Yes
		Mean age: 42.3 (SD 6.09)			

### Usability testing procedure

All tests were conducted via remote digital media by the lead author (RY). The virtual meetings were password protected and the meeting room was locked once the participant had entered the system. A short familiarisation session was scheduled to ensure that the remote technology could be accessed by each participant. The Zoom media ‘remote control’ function was synched with a screen share of the adobe interface. The participants were supported through activation of the remote-control mouse icon and supported in briefly navigating through the virtual GUI to ensure that they could activate the functions and view the interface from their selected device. Test one was scheduled during each familiarisation session. The familiarisation meeting, test one and test two were recorded directly to the lead author’s device into a secure digital storage system at the host university.

Test one evaluated the usability of version 1 of the GUI and comprised three specific tasks (1.1, 1.2, 2.0) in the ‘Quick Start’ programme (Table 3). Participants were asked to verbalise their thoughts about navigating through the GUI using a ‘think-aloud’ technique [31]. Alongside the ‘think aloud’ data task completion rates and task duration data were collected. Each task was completed twice. During the first attempt at each task, participants were encouraged to ‘think aloud’ as they navigated through the interface and identified the icons which would enable task completion. They were prompted to explain their decisions and verbally share their experience of navigating the interface. The second attempt was conducted in silence and participants were required to directly navigate through the task under timed conditions.

**Table 3:** Usability tasks

Version 1 and 2	Completion criteria	Timed components
Task 1.1	Access quick start Select 6 min Activate virtual exercise programme	1.1: Time lapsed from login and virtual exercise
You want to do a 6-min workout in the ‘quick start’ programme		
Task 1.2		1.2: Time lapsed from login to opening the results menu
You will view your results at the end of the exercise	View results	
Task 2.0	Access quick start Select 4 min Activate virtual exercise Activate ‘help’ icon	2.0: Time lapsed from login to opening assistance called menu
You want to do a 4-min workout in ‘quick start.’ As the machine starts to move you realise your hand is not secured to the moving component and you decide to call for help		
Version 2 only		
Task 3.1	Access ‘my programme’ Access ‘baseline assessment’ Activate virtual exercise	3.1: Time lapsed from login to virtual exercise
You want to complete a baseline assessment in the ‘my programme’ area. Assistance is available		
Task 3.2	Increase target intensity View results	3.2: Time lapsed from login to opening the results menu
You decide that you would like to increase the target intensity during exercise and view your results on completion		
Task 4.1	Access ‘my programme’ Select ‘hilly’ or ‘steady’ Activate virtual exercise	4.1: Time lapsed from login to virtual exercise
Please choose either the ‘hilly’ or ‘steady’ option in the ‘my programme’ area		
Task 4.2		4.2: Time lapsed to opening the results menu
You decide that you would like to decrease the target intensity during exercise and view results on completion	Decrease target intensity View results	

**Fig. 4:**
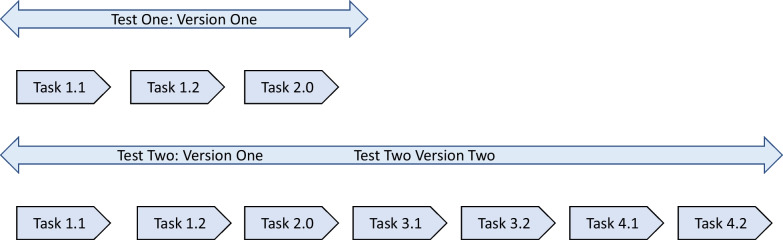
Timeline to represent tasks conducted on version one and version two: The first three tasks were conducted on versions one and two. The final four tasks were specific to the new submenus created within version 2

Test two was conducted on the same sample of participants and. scheduled between four to six weeks after test one and evaluated the usability of version 2 of the GUI. Tasks 1.1, 1.2 and 2.0 were repeated and four additional tasks (3.1, 3.2, 4.1, 4.2) were introduced to evaluate the extended ‘my programme’ submenu of the GUI. The purpose of repeating the test one tasks was to establish whether the changes implemented between version 1 and version 2 affected the usability of the GUI. In order to optimise consistency of testing conditions, each task was repeated twice, with the first attempt being a ‘think aloud’ walkthrough of the GUI and the second attempt a timed test conducted in silence (Fig. 4).

The research team were cognisant of ensuring a positive participant experience throughout all testing procedures. The lead author advised that the tasks were not intended to test the capabilities of the participant and that any difficulties encountered whilst completing the tasks reflected shortfalls in the design of the GUI. The lead author is an experienced neurological physiotherapist with knowledge of the communication and processing impairments which can occur following stroke. Verbal instructions and prompts were adapted according to responses from each participant and rest time was offered between each task.

### Usability observation form

Test one and test two were audio–video recorded to enable identification of usability issues, record task completion and task duration. A usability observation form was used to document all findings (Additional file 1). Cursor tracking was observed on the video footage of each virtual test; errors, hesitation or delays in navigation through the GUI were documented as a usability occurrence. The ‘think aloud’ data were initially summarised onto the usability observation form by the lead author. Four of the recordings alongside the respective usability observation forms were sense checked by a second member of the research team (NS). Discussion between RY and NS led to agreement that the ‘think aloud’ data would be transcribed verbatim onto the usability observation form to ensure the user experience was fully captured. Narrative which indicated user uncertainty, hesitation or dissatisfaction with the GUI was documented as a usability occurrence.

### Participant satisfaction

The PSSUQ was selected to capture participants’ experience of the GUI on completion of each test. The PSSUQ is a 16-item standardised questionnaire devised to measure users’ perceived satisfaction of a software system (Tables 4, 5). The PSSUQ has concurrent validity [45], very high scale and subscale reliability and construct validity [46]. Participants were required to complete a 7-point Likert scale with responses ranging from strongly agree (1) to strongly disagree (7) (Table 5). An overall mean score is calculated from PSSUQ responses plus individual scores for three subsections: system usefulness, information quality and interface quality (Table 4). Lower mean scores indicate higher user satisfaction [40]. Participants were issued with an on-line version of the questionnaire at the end of each test and requested to complete it and submit responses within 24 h.

**Table 4:** Post-Study System Usability Questionnaire

Subsection	Questions
System usefulness	1. Overall, I am satisfied with how easy it was to use this system
	2. It was simple to use this system
	3. I was able to complete the tasks and scenarios quickly using this system
	4. I felt comfortable using this system
	5. I believe I could be productive quickly using this system
	6. I believe I could become productive quickly using this system
Information quality	7. The system gave error messages that clearly told me how to fix problems
	8. Whenever I made a mistake using the system I could recover easily and quickly
	9. The information provided with this system was clear
	10. It was easy to find the information I needed
	11. The information was effective in helping me complete the tasks and scenarios
Interface quality	12. The organisation of information on the systems screens was clear
	13. The interface of this system was pleasant
	14. I liked using the interface of this system
	15. This system has all the functionalities and capabilities I expect it to have
	16. Overall, I am satisfied with this system

**Table 5:** Post-Study System Usability Questionnaire Scoring Scale

On a scale from Strongly Agree to Strongly Disagree, please rate the following statements								
(Positive Statement)	1	2	3	4	5	6	7	NA

### Data analysis

Descriptive and inferential statistics were conducted in Excel (Microsoft) and SPSS (IBM version 28.0.0.).

#### Usability issues

Usability occurrences recorded on the usability observation forms were collated to identify the total number of incidents detected through cursor tracking and ‘think aloud’ data on version 1 and version 2 of the GUI. Usability incidents which recurred across participants were clustered to develop a definitive list of usability issues. The identified usability issues were coded according to four a-priori categories developed during stages one and two of the research programme [13, 20]. The categories were; (1) system safety; (2) operational efficiency; (3) programme effectiveness; and (4) user engagement.

To determine which usability issues required prioritisation, the frequency of occurrence was collated and severity was scored. Frequency was recorded on a modified user by problem matrix (Table 6) [31]. Total issue occurrence was summated to enable comparison between the user streams and incidence of problems on versions 1 and 2 of the GUI.

**Table 6:** User by problem matrix

Usability issues category	Participant code																				Fr*	S **
	EU1	EU2	EU3	EU4	EU5	EU6	EU7	EU8	EU9	EU 10	PU1	PU2	PU3	PU4	PU5	PU6	PU7	PU8	PU9	PU 10		
Safety																						
Operational																						
Programme effectiveness																						
User engagement																						

*Frequency of problem

**Severity of problem

The problem severity scale developed by Dumas and Redish [31] was adapted to identify features which may cause risk of injury, impede programme effectiveness or reduce user engagement. Table 7 indicates the adapted categories in italics. All detected usability issues were scored to determine severity.

**Table 7:** Problem severity scale

Level 1	Prevents task completion May lead to user injury May cause programme to be ineffective May cause user disengagement
Level 2	Creates significant delay or frustration Significantly impedes programme effectiveness
Level 3	Problems have minor effect on usability May have minor effect on programme effectiveness May cause minor user uncertainty
Level 4:	Subtle and possible enhancements/suggestions

Descriptive analysis of the user by problem matrix was conducted to examine the pattern of usability issues across the a-priori categories and compare sequential versions of the GUI.

Two members of the research team (RY and AH) discussed each usability issue, considering the frequency and severity to determine which usability issues would be addressed in the iteration of version 2 of the GUI. Usability issues with a severity score of four were automatically addressed.

#### Task completion

Task completion was defined as navigation through all required submenus within the GUI to access the exercise programme, user performance or assistance request stipulated in the task descriptor. No time limit was applied. Instances in which a participant made an error but was able to self-correct and navigate to the intended menu were recorded as task completion. Task completion data were recorded and collated on the usability observation form.

#### Task duration

Shapiro-Wilks tests (significance 0.05) were conducted on task time to determine normal distribution. Calculation of the task duration geometric mean mitigated for the positively skewed data distribution which is a common occurrence with timed tasks [40]. One sample T-Tests were conducted on the geometric means calculated for tasks 1.1 and 4.1 to determine the probability of 95% of users commencing exercise within the benchmark target of 25 s.

Two-tailed T-Tests are considered robust to the positive skew associated with task duration data and log transformation is not required [40]. Two-tailed paired t-tests were conducted on the mean difference scores between version 1 and version 2 for tasks 1.1, 1.2 and 2.0 to detect any statistically significant difference in repeated task times. Independent T-Tests were conducted on all task time data to detect any statistically significant difference in completion times recorded between the EU and PU groups.

#### User satisfaction

Shapiro-Wilks tests (significance 0.05) were conducted on task time to determine normal distribution. Total PSSUQ scores were analysed in addition to analysis of the individual sub-sections. An independent samples T-Test was conducted on the difference in scores between the user streams for version 1 and version 2 of the GUI.

## Results

The results are presented in alignment with the underpinning objectives of the study.

### Evaluate version 1 of the GUI

The total occurrence of usability issues detected and recorded during the examination of version 1 was 100. Each incident was described and coded to the relevant a-priori category which enabled identification of recurrent usability problems. The distribution of usability incidents across the four categories on version 1 was 24% safety, 28% operational, 22% programme effectiveness and 26% user experience.

Twenty-two different usability issues were identified during the testing of version 1 (Table 8), a detailed listing of these can be accessed in the supplementary materials 2.0. Each problem was analysed by two members of the research team (RY, AH) and the decision regarding whether to directly address the problem in the iteration of version 2 was determined by the issue frequency, severity and feasibility of adapting the underpinning technology.

**Table 8:** Usability issues according to category

Category	Number of detected usability problems	Number of usability occurrences
Safety	4	24
Operational	8	28
Programme effectiveness	6	22
User engagement	4	26

#### Safety

**Fig. 5:**
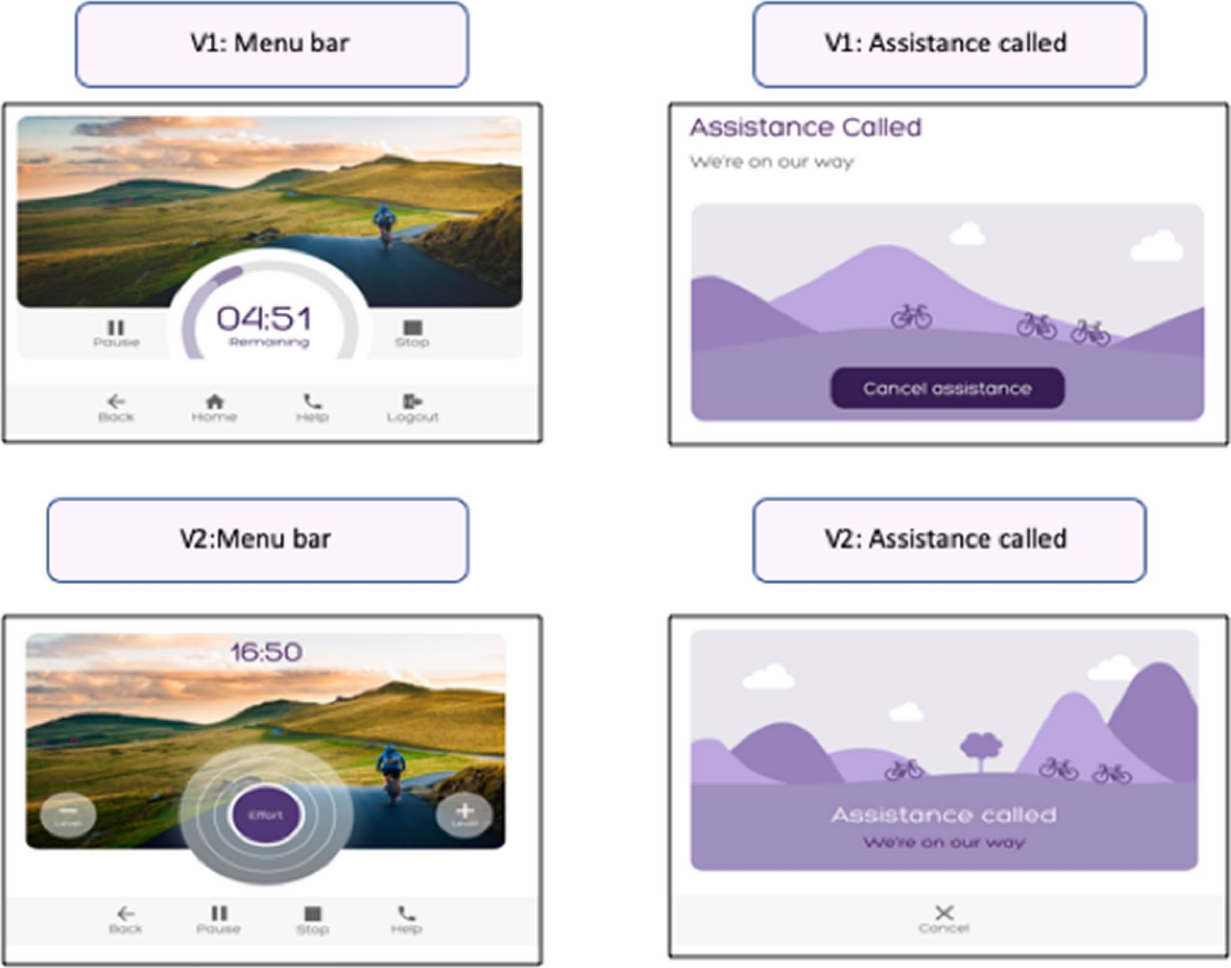
Safety problems addressed: Stop and pause icons were added to the menu bar and the ‘assistance called’ message was centralised

Features which could lead to the machine commencing or sustaining unintended movement were identified as a safety risk, alongside difficulties associated with requesting help. The usability tests completed on version 1 of the GUI indicated that the ‘help’ icon was not visible enough and the ‘assistance called’ text was easy to miss. Ten participants reported feeling unsure about the difference between the stop/pause/help functions visible during live exercise. To address these problems, the menu bar visible during the live exercise phase of the programme was reconfigured to display distinct icons for pause, stop and help. The icons were slightly larger and the ‘help’ icon was positioned on the end of the menu bar. On the ‘assistance called’ page, the ‘cancel’ icon was relocated to the bottom of the page with the ‘assistance called’ text centralised (Fig. 5).

#### Operational

**Fig. 6:**
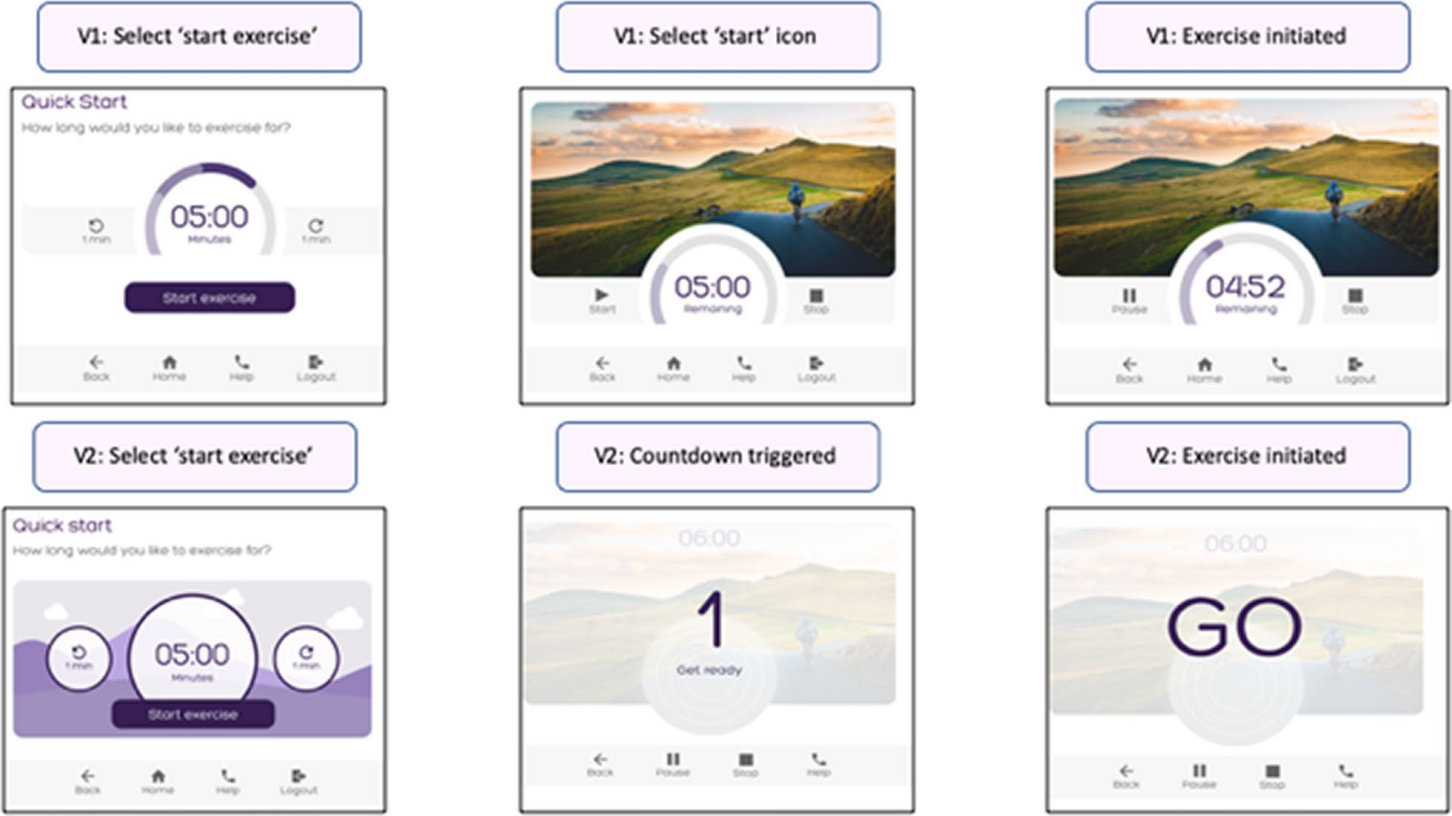
Operational problems addressed: On version 2, activation of the ‘start exercise’ icon triggered a countdown to commencement of movement avoiding the need for a second click on the ‘play’ icon

Usability issues which could lead to a delay in users operating the equipment or cause them to require frequent guidance from support staff were coded within the operational category. Eight operational problems were identified on version 1; the most frequently occurring usability problem was associated with the duplication of activating the ‘start/play’ icons to commence exercise. Delays in identifying the ‘start’ icon were observed amongst nine participants. Five participants across both groups verbally reported that the repeated clicking to activate the machine could cause frustration or confusion. These issues were directly addressed in version 2 of the GUI. Instead of clicking a ‘start’ and then ‘play’ icon to initiate exercise, activation of ‘start exercise’ triggered a three second countdown with no repeated clicks required. The background to the ‘select duration’ page was adjusted to ensure that the functional icons were distinct (Fig. 6). Six operational issues with low frequency and severity scores were not addressed (Additional file).

#### Programme effectiveness

**Fig. 7:**
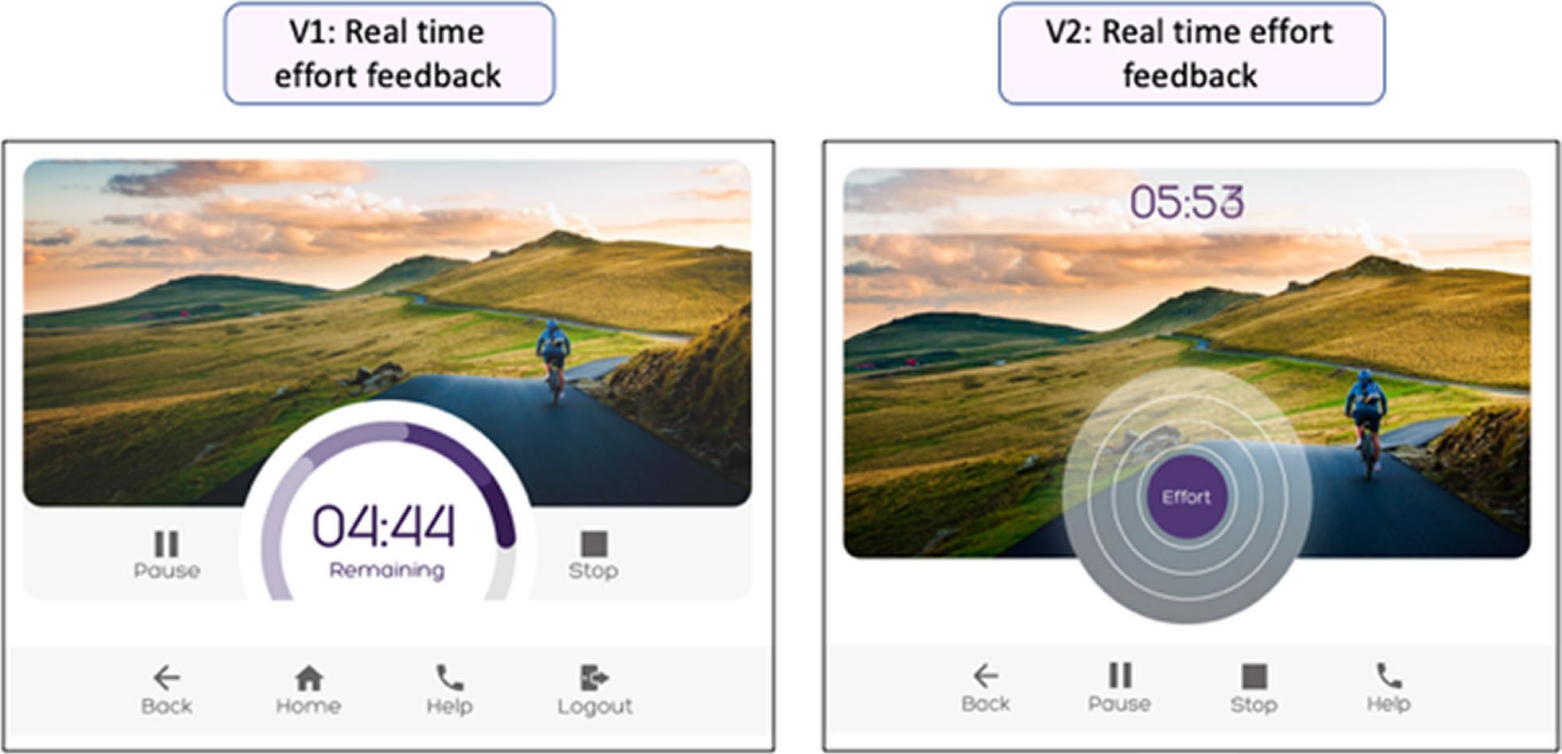
Programme effectiveness problems addressed: The effort biofeedback was re-designed on version 2. The expanding circle replaced the dial used on version 1

The programme effectiveness category identified those problems associated with the GUI which had the potential to impede users in engaging in an optimal intensity of exercise or quality of movement. Real time feedback regarding intensity of effort was a pivotal feature of the co-designed GUI; however, usability testing of version 1 indicated that 13 of the 19 participants misinterpreted the effort feedback dial. The real time visualisation of detected effort was identified as a priority for amendment in version 2 of the GUI. The redesign introduced an expanding and contracting balloon as an alternative to the feedback dial visualised in version 1. (Fig. 7).

#### User engagement

**Fig. 8:**
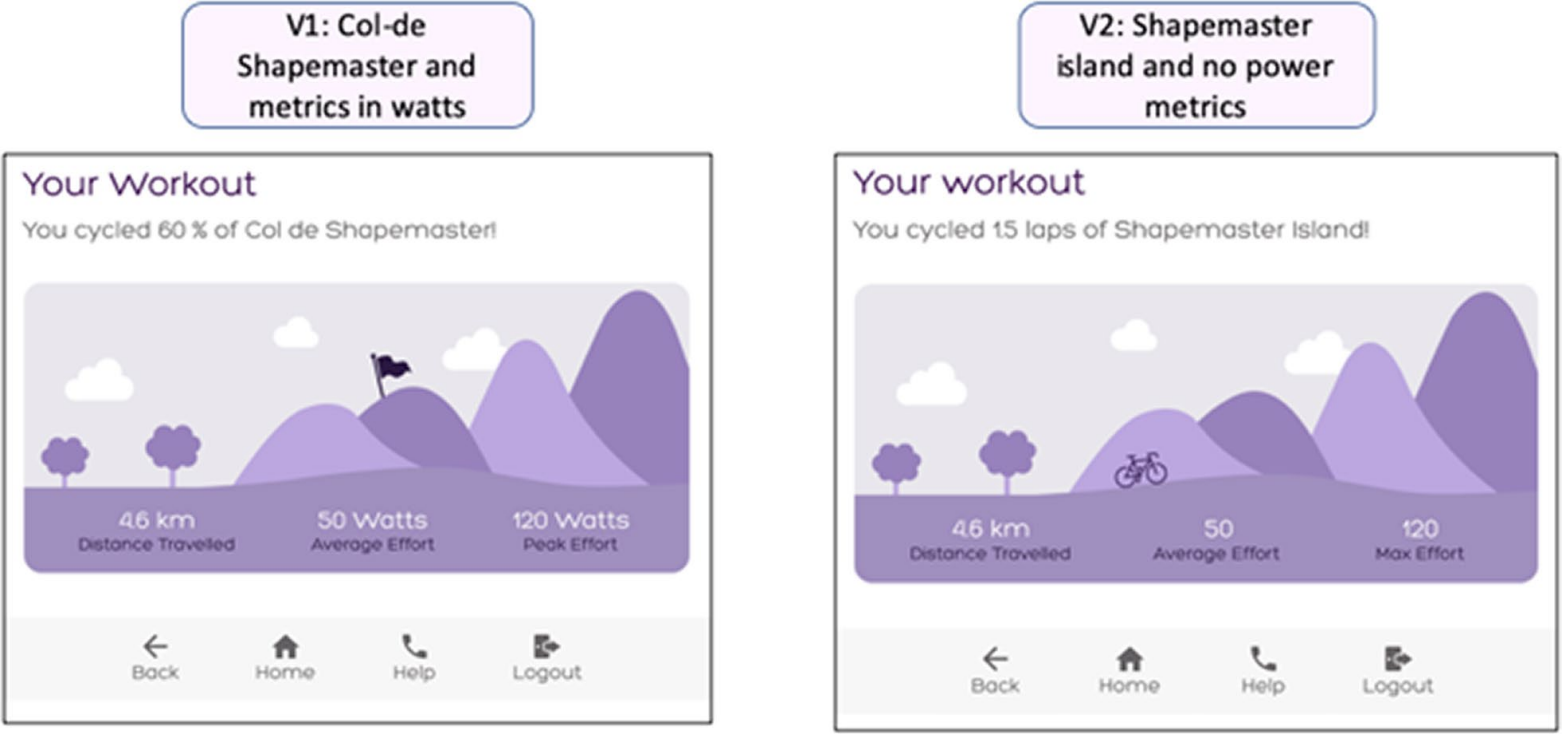
User engagement problems addressed: The concept of ‘Col-de Shapemaster’ was replaced by ‘Shapemaster Island’ and watts were removed from the metric details

Concerns regarding clarity of performance results and motivational features were categorised into this section. Usability testing of version 1 indicated that nine participants did not understand Watts as a performance metric. Eight participants reported that the concept of cycling up a ‘col de Shapemaster’ was not meaningful and two participants shared that the still image was uninspiring. Version 2 of the GUI displayed standalone numbers and the ‘col de Shapemaster’ concept was replaced by ‘Shapemaster Island.’ (Fig. 8).

#### Task completion rates and task duration

Analysis of task completion and duration enabled the research team to quantify the usability of the GUI in the context of specific tasks aligned with its projected purpose. During the testing of version 1, EU7 experienced difficulties with remote control connectivity causing the completion times for tasks 1.1 and 1.2 to be invalid and not included in the descriptive analysis; task 2.0 was abandoned. Task completion and duration data are detailed in Table 9.

**Table 9:** Version 1 task duration and completion

Participant	Task 1.1 (sec)	Task 1.2 (sec)	Task 2 (sec)
EU1	12	51	31
EU2	20	63	30
EU3	19	56	32
EU4	27	67	26
EU6	33	77	38
EU7	280 *	380*	Terminated
EU8	16	55	19
EU9	26	66	32
EU10	21	59	13
PU1	9	48	24
PU2	20	57	25
PU3	16	55	25
PU4	21	59	20
PU5	21	55	28
PU6	15	53	18
PU7	12	51	19
PU8	11	48	34
PU9	11	47	22
PU10	13	57	22
Range	9–33	47–77	18–38
Median duration	17.5	55.5	25
% Task completion	100%	100%	100%

*Invalid data due to connectivity

The completion rate for all tasks was 100% except for Task 2.0 for EU7 which was attributed to failed connectivity rather than navigation through the GUI.

The benchmark duration for Task 1.1 was 25 s which was the maximum duration from opening the GUI to commencing exercise stipulated by representative commercial operators. For this analysis, the EU and PU group data were analysed individually as the intention was for EUs to operate the GUI independently in a real-world setting.

Calculation of the geometric mean using log transformation of task duration data generated a better estimate of the central values and has less error or bias than the standard mean for small samples of usability data [40]. One tailed T-tests were conducted on the geometric means calculated from Task 1.1 data recorded from version 1 of the GUI for the EU and PU groups to determine the probability of 95% of users achieving the benchmark target (Table 10).

**Table 10:** Version 1 task 1.0 benchmark comparison

	Geometric Mean (SD) in seconds	Benchmark in seconds	P-value	Probability
EU Group	18.4 (1.48)	25	P = 0.03	96.62%
PU Group	13.7 (1.35)	25	P = 0.0001	99.99%

#### User satisfaction

All participants who completed the usability test on version 1 (n = 19) submitted PSSUQ responses. Analysis of PSSUQ scores indicated high levels of user satisfaction across both user groups and favourable comparison with PSSUQ normative data. Due to limitations associated with published normative values, inferential analysis would not have represented a meaningful comparison [38]. The ‘information quality’ subsection attained the lowest satisfaction scores across both user groups and this pattern is mirrored in the published normative data [40] (Table 11).

**Table 11:** PSSUQ data comparing PU and EU results

PSSUQ section	PU group Version 1 (n = 10)	EU group Version 1 (n = 9)	Whole group Version 1 (n = 19)	PSSUQ norms
	Mean (SD)	Mean (SD)	Mean (SD)	Mean
System usefulness	1.52 (0.31)	1.38 (0.36)	1.44 (0.33)	2.80
Information quality	1.85 (0.24)	1.61 (0.62)	1.72 (0.49)	3.02
Interface quality	1.60 (0.47)	1.33 (0.48)	1.45 (0.48)	2.49
Overall score	1.63 (0.24)	1.44 (0.46)	1.52 (0.38)	2.82

Lower score indicates higher satisfaction [27]

The scores submitted by the EU group were slightly lower than the PU group indicating greater satisfaction amongst the EU group. An independent samples T-Test was conducted on the difference in scores between the two groups, no statistically significant difference in satisfaction between the user groups was detected (P = 0.296, confidence interval − 0.19 to 0.58).

### Develop and evaluate an extended version 2 of the GUI

#### Development of version 2

Version 2 of the GUI addressed eight of the usability issues identified during the testing of version 1 and these are detailed in Table 12.

**Table 12:** Summary of usability problems addressed

Category	V1 problem	V2 amendment
Safety	Help button not visible enough	Help icon more centrally positioned on menu bar
	Assistance called message not visible enough	Assistance called message centralised
	Distinction between stop/pause/help functions not clear	Menu bar reformatted
Operational	Repeated clicks to start exercise	‘Start exercise’ icon triggered a countdown to exercise
	Select duration/start exercise icons not visible enough	Background and icon boundaries amended to be more distinct
Programme effectiveness	Effort detection dial misinterpreted	Effort detection displayed as an expanding balloon
User experience	Performance metrics (watts) not understand by users	Standalone numbers displayed
	‘Col-de-Shapemaster’ concept not meaningful	‘Shapemaster Island’ concept introduced

**Fig. 9:**
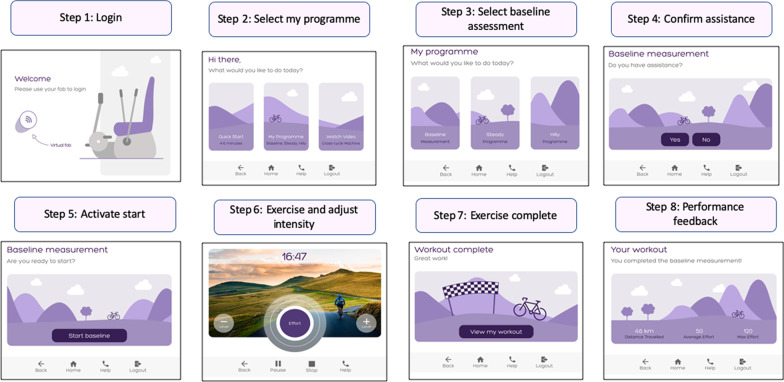
Graphical User Interface version 2 baseline assessment menu: The login submenu and programme selection were developed from version 1. Steps 3–8 illustrate the ‘baseline assessment’ function

Version 2 also included an extended range of programme options underpinned by an individualised baseline assessment. The intention was to develop a tailored prescription of exercise at an optimal intensity for the individual user. The ‘baseline assessment’ programme would be completed with supervision from an exercise or rehabilitation professional to ensure an appropriate intensity and duration of exercise (Fig. 9).

**Fig. 10:**
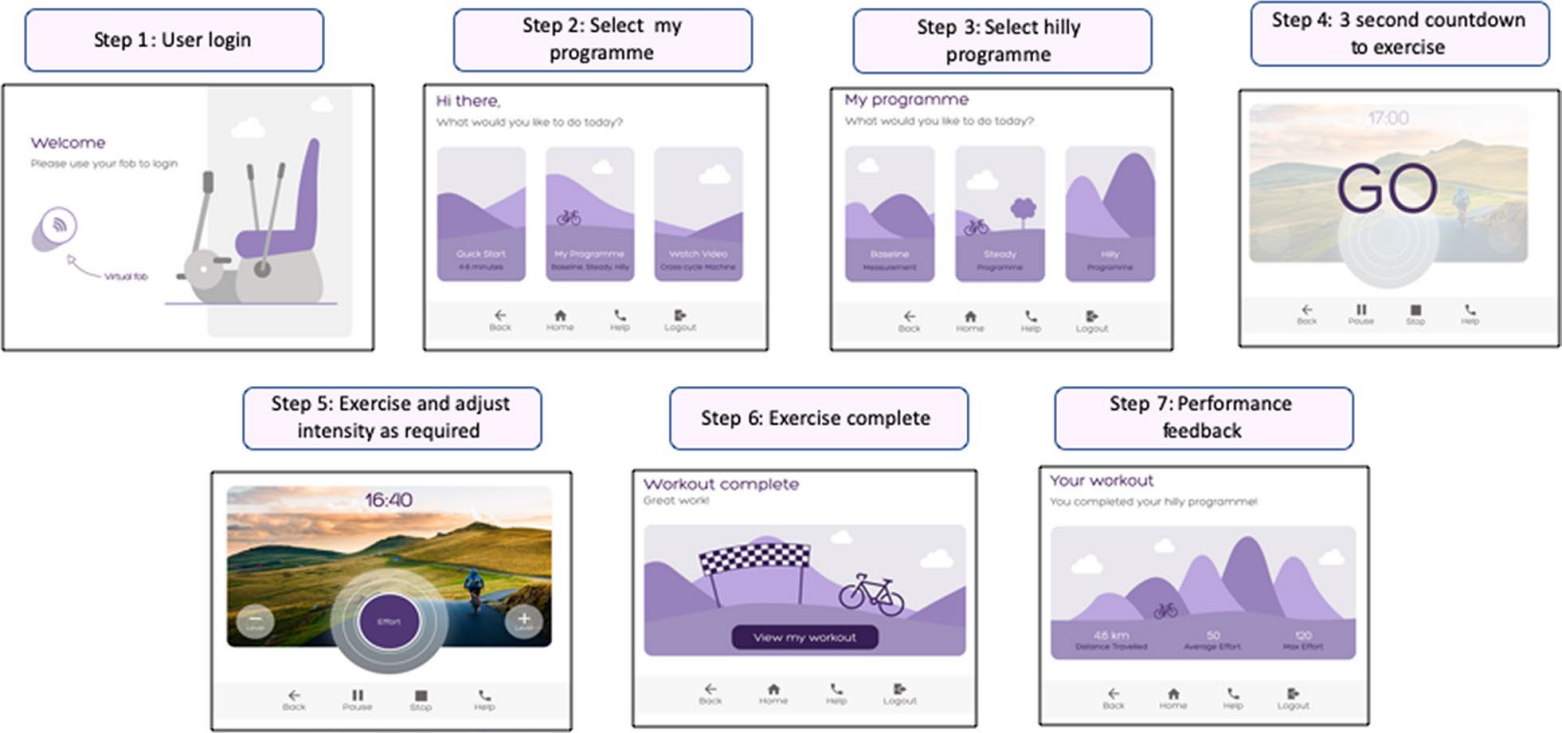
Graphical User Interface Version 2 hilly exercise programme menu: The white margin outside the purple circle indicated the target effort for the user

The ‘my programme’ menu also included the choice of either a ‘steady’ or ‘hilly’ interval programme. The target intensity was indicated by a white balloon, with detected purple effort expanding within it (step three in Fig. 10).

#### Evaluation of version 2

The total occurrence of usability issues detected during the evaluation of version 2 was 86. Each incident was described and coded to the relevant a-priori category which enabled identification of arising and recurrent usability problems. The distribution of usability incidents across the four categories on version 2 was 12% safety, 29% operational, 40% programme effectiveness and 19% user experience (Table 13). Two recurrent issues identified during testing of version 1; identification of the ‘help’ icon and interpretation of effort detection feedback. 24 new usability issues were identified.

**Table 13:** Version 2 usability incidents

Category	Number of recurrent usability problems	Number of new usability problems	Number of usability occurrences
Safety	1	3	10
Operational	0	5	25
Programme effectiveness	1	10	35
User experience	0	6	16

#### Safety

Usability testing on version 2 of the GUI indicated that identification of the ‘help’ icon remained an issue for two participants and three new usability problems were detected. Four participants reported that a new countdown feature did not allow enough time to prepare for machine movement. One PU participant was concerned that the plus and minus icons on the live exercise page could be mistaken for speed adjustment and three participants were concerned that users would proceed without assistance during a baseline assessment.

#### Operational

Testing of version 2 of the GUI indicated that the operational problems observed in version 1 did not recur. However, the introduction of the extended ‘my programme’ area of the GUI did create five new usability problems associated with the new features. The concept of a baseline assessment, intended for new users or people wishing to review their progress, created confusion amongst PUs and EUs It was suggested that substantial explanation and support would be needed to support users in navigating this programme option. The omission of duration selection option for the ‘hilly’ or ‘steady’ workout options was identified by five participants and has the potential to cause operational disruption if not amended in future iterations.

#### Programme effectiveness

Usability tests completed on version 2 indicated that the new iteration of the real time effort feedback was much clearer than version 1, with only one participant (EU9) expressing uncertainty. However, the new features introduced into the ‘my programme’ area generated a range of new usability issues. The most frequently occurring problem was associated with uncertainty regarding the purpose of the white circle which was intended to indicate the target intensity. The other problems were associated with the intensity selection function, absence of temporal tracking, speed selection and heart rate feedback (Additional file 2).

#### User experience

The unquantified numbers on the results page raised a concern by six participants across the PU (3) and EU (3) groups who reported that a metric was needed. Three different participants, two from EU and one from PU groups, observed that the ‘Shapemaster Island’ concept was not consistently embedded across the menus of the GUI. The importance of feedback regarding symmetry of feedback was expressed by two PU participants and two different PU participants noted that the intensity level was not included in the results page.

Usability issues with a severity score of 4 or occurrence greater than 25% are summarised in Table 14 and will be considered for amendment in the next iteration of the GUI.

**Table 14:** Serious usability issues identified on version 2

Category	V2 problem	Proposed amendment
Safety	Countdown to machine starting too short	Increase from 3 to 5 s
	Potential for user to proceed into baseline assessment without assistance	Create a security code for this area of the submenu
	Help icon not visible enough	Explore colour options, e.g. red icon
Operational	Concept of baseline assessment unclear	Address through staff training and information video
	Purpose of assistance for baseline assessment unclear	Address through staff training and information video
	Did not notice the ‘measurement/programme’ subtext	Increase size and darken colour of font
	Cannot select duration in ‘my programme’ menu	Add this function in the same format as used in ‘quick start’ menu
Programme effectiveness	Purpose of target intensity circle not obvious	Include explanation in information video and review biofeedback graphics
	Selected intensity not displayed	Add the selected number to the ‘live exercise’ menu
	No feedback if you press the plus or minus icons for intensity	Display intensity number selected
User experience	Unquantified metrics do not represent meaningful feedback on performance	Continue user involvement programme to identify optimal metrics

#### Task completion and duration

The completion rate for all tasks was 100%. Individual duration of each task for each participant is summarised in Table 15.

**Table 15:** Version 2 task completion and duration

Participants	Task 1.1 (sec)	Task 1.2(sec)	Task 2 (sec)	Task 3.1 (sec)	Task 3.2 (sec)	Task 4.1 (sec)	Task 4.2 (sec)
EU1	12	52	21	17	56	10	48
EU2	14	64	25	34	75	47	88
EU3	10	52	23	18	65	16	46
EU4	25	72	49	31	75	19	66
EU6	34	77	45	29	71	17	58
EU7	34	79	34	15	53	34	75
EU8	16	57	20	18	58	13	51
EU9	20	89	43	29	69	15	45
EU10	16	54	16	20	54	12	46
PU1	13	49	16	21	56	12	49
PU2	11	49	23	20	63	13	50
PU3	14	54	20	20	59	12	51
PU4	15	53	26	21	58	14	52
PU5	W/D*	W/D*	W/D*	W/D*	W/D*	W/D*	W/D*
PU6	15	53	19	20	59	15	48
PU7	14	52	17	19	63	12	54
PU8	12	52	22	19	65	9	48
PU9	13	53	18	22	60	12	48
PU10	14	52	20	23	67	12	51
Range (sec)	11–34	49–89	18–49	17–34	53–75	9–47	46–88
Median duration (sec)	14	53	21.15	20	61.5	13	50.5
% Task completion	100%	100%	100%	100%	100%	100%	100%

*Participant withdrawn

Repeated analysis of achievement of the 25 s benchmark duration for task 1.1 was repeated on the task duration data recorded during the testing of version 2. The geometric mean using log transformation of task duration data was calculated for each user group and one tailed T-tests were conducted. The results summarised in Table 16 indicate the probability of 95% attainment of the target benchmark across both user groups.

**Table 16:** Task 1.1 benchmark comparison

	Geometric mean (SD) in seconds	Benchmark in seconds	P-value	% Achievement
EU Group	17.1 (1.48)	25	P = 0.0153	98.46%
PU Group	13.3 (1.10)	25	P = 0.0001	99.99%

The baseline assessment programme evaluated during Tasks 3.1 and 3.2 required a user induction or formal review which would be supervised, therefore the benchmark target duration was not applicable. However, Task 4.1 was intended to evaluate independent navigation through the GUI and the 25 s benchmark target was applicable. Analysis of user group attainment of this is detailed in Table 17.

**Table 17:** Task 4.1 benchmark comparison

	Geometric Mean (SD) in seconds	Benchmark in seconds	P-value	% Achievement
EU Group	19.3 (1.55)	25	P = 0.074	92.53%
PU Group	12.2 (1.16)	25	P = 0.0001	99.99%

The probability of attaining the 25 s benchmark amongst the EU group was below 95% indicating that this programme option may have the potential to cause operational disruption due to user delay.

#### User satisfaction

All participants who completed the usability test on version 2 submitted PSSUQ responses, however, two data sets from the PU group were discarded due to a technical issue with the survey software. The ‘information quality’ subsection attained the lowest satisfaction scores amongst the EU group, whereas ‘interface quality’ was the aspect of lowest satisfaction amongst the PU group. Comparison with normative PSSUQ data indicated good levels of user satisfaction (Table 18).

**Table 18:** User satisfaction scores

PSSUQ section	PU group Version 2 (n = 7)	EU group Version 2 (n = 9)	Whole group Version 2 (n = 15)	PSSUQ norms
	Mean (SD)	Mean (SD)	Mean (SD)	Mean
System usefulness	1.26 (0.18)	1.23 (0.27)	1.24 (0.23)	2.80
Information quality	1.49 (0.31)	1.69 (0.69)	1.61 (0.55)	3.02
Interface quality	1.57 (0.51)	1.27 (0.31)	1.40 (0.42)	2.49
Overall score	1.41 (0.30)	1.40 (0.32)	1.41 (0.30)	2.82

The scores indicated by the EU group were slightly lower than the PU group indicating greater satisfaction amongst the EU group. An independent samples T-Test was conducted on the difference in scores between the two groups, no statistically significant difference in satisfaction between the user groups was detected (P = 0.827, confidence interval − 0.30 to 0.37).

### Comparison of the usability of version 1 and version 2

Direct comparison between the ‘quick start’ programme on version 1 and version 2 aimed to evaluate differences recorded pertaining to problem occurrence, type of usability issues detected and performance of tasks 1.1, 1.2 and 2.0. The extended menus explored on version 2 created a new user experience and therefore statistical comparison of user satisfaction as reported in the PSSUQ was not explored.

#### Usability issues

Five usability issues were identified on the ‘quick start’ submenu on version 2, compared with 22 on version 1. Two of the issues identified on version 2 were recurrent; visibility of the ‘help’ icon and clarity of the effort detection biofeedback. However, the frequency of problem occurrence was lower, with one participant reporting difficulty associated with interpretation of the biofeedback on version 2 compared with 13 participants during the testing of version 1. Three new usability issues were associated with the changes made between version 1 and version 2. The countdown feature was considered too short and potentially unsafe by four participants; six participants did not like the absence of performance metrics and three participants reported that the ‘Shapemaster Island’ theme was inconsistent.

#### Task performance

With the exception of the connectivity issues which affected EU7 during the testing of version 1, there was 100% task completion for tasks 1.1, 1.2 and 2.0 across versions one and two of the GUI.

Shapiro-Wilks (significance 0.05) tests conducted on task duration data indicated normal distribution. Log transformation of raw task duration data is not required for comparison between mean values as two-tailed t-tests are considered robust to the positive skew associated with this type of data set [27]. Paired two tailed T-tests were performed on the mean difference between version 1 and version 2 completion times to detect any statistically significant difference between version 1 and version 2 task duration data [27] (Table 19). Participants with incomplete task duration data sets (PU5, EU7) were excluded from this stage of analysis.

**Table 19:** Mean difference in task duration scores (v1–v2)

EU group (n = 8)	Mean (SD) difference v1–v2 (secs)	P value (2 tailed) Confidence interval	PU group (n = 9)	Mean (SD) difference V1–V2 (secs)	P value (2 tailed) Confidence interval
Task 1.1	− 3.37 (3.62)	P = 0.03* (0.35–6.39)	Task 1.1	− 0.77 (4.20)	P = 0.59 (− 2.46 to 4.01)
Task 1.2	+ 2.87 (8.74)	P = 0.38 (− 10.1 to 4.41)	Task 1.2	− 1.11 (4.64)	P = 0.49 (− 2.46 to 4.69)
Task 2.0	+ 2.62 (11.08)	P = 0.52 (− 11.87 to 6.62)	Task 2.0	− 3.11 (5.13)	P = 0.10 (− 0.84 to 7.06)

Duration was significantly faster for Task 1.1 on version 2 of the GUI compared to version 1 amongst the EU group (p = 0.03). A non-significant increase in duration of tasks 1.2 and 2.0 on version 2 was recorded amongst the EU group. A non-significant decrease in all task duration between Versions 1 and 2 amongst the PU group was recorded.

### Analyse usability as experienced by EU and PU participants

Comparison between the EU and PU groups aimed to ensure that the GUI was accessible and intuitive for use by PwS and supporting professionals. Detection of significant differences in task performance and user satisfaction would enable the team to identify features on the GUI which may require specific amendment. The occurrence of usability problems and task performance data were analysed to detect any differences between the usability as experienced by the two user groups. The distribution of problem occurrence across user groups on the two versions of the GUI is summarised in Table 20.

**Table 20:** Frequency of problem occurrence

User group	V1 safety	V2 safety	V1 operational	V2 operational	V1 programme effectiveness	V2 programme effectiveness	V1 User experience	V2 User experience	Total
EU	9	4	11	10	9	18	11	9	81
PU	15	6	17	15	13	17	15	7	105
Total	24	10	28	25	22	35	26	16	186

During the testing of version 1, 40 usability incidents were detected amongst the EU group compared with 60 incidents amongst the PU group. The PU group were more likely to encounter or identify concerns regarding the safety, operational efficiency and effectiveness of the system when compared with the EU group. On version 2, the distribution of usability incidents was 41 for the EU group, compared with 45 amongst the PU group. Aspects of the extended ‘my programme’ menu on version 2 were unclear to both user groups, particularly the target intensity circle and selection of programme intensity. This accounted for the high occurrence of usability issues amongst PU and EU participants in the programme effectiveness category of version 2.

Task duration was compared between the EU and PU groups to detect any statistically significant differences in usability experienced by PwS. Independent two-sided T-Tests were conducted to compare mean completion time between the EU and PU group (Table 21).

**Table 21:** Comparison of task duration between professional and expert users (version 1)

Task 1.1		Task 1.2		Task 2.0	
EU Mean (SD) (sec)	PU Mean (SD) (sec)	EU Mean (sec)	PU Mean (sec)	EU Mean (sec)	PU Mean (sec)
21.7 (6.67)	14.2 (4.14)	61.7 (4.55)	53.0 (4.55)	27.6 (2.84)	23.2 (1.58)
P = 0.018* (− 13.5 to − 1.53)		P = 0.023* (− 16.0 to − 1.44)		P = 0.204 (− 11.5 to 2.76)	

The PU participants were significantly quicker than the EU participants to complete Task 1.2 on both versions of the GUI. Although the PU participants were quicker to complete Tasks 1.1 and 2.0, the difference was only statistically significant on Task 1.1 in version 1. The PU participants were quicker to complete Tasks 3.1, 3.2, 4.1 and 4.2 but the difference between the user groups did not reach statistical significance (Table 22).

**Table 22:** Comparison of task duration between professional and expert users (version 2)

Task 1.1		Task 1.2		Task 2.0	
EU Mean (SD) (sec)	PU Mean (SD) (sec)	EU Mean (sec)	PU Mean (sec)	EU Mean (sec)	PU Mean (sec)
18.3 (7.8)	13.4 (1.3)	64.4 (13.5)	51.8 (1.7)	30.2 (13.1)	20.1 (3.1)
P = 0.06 (− 11.51 to 1.65)		P = 0.033* (− 24.1 to − 1.36)		P = 0.067 (− 21.1 to 0.99)	

Task 3.1		Task 3.2		Task 4.1		Task 4.2	
EU Mean (SD) (sec)	PU Mean (SD) (sec)	EU Mean (SD) (sec)	PU Mean (SD) (sec)	EU Mean (SD) (sec)	PU Mean (SD) (sec)	EU Mean (SD) (sec)	PU Mean (SD) (sec)
23.4 (7.19)	20.5 (1.33)	64.0 (8.9)	61.1 (3.5)	17.0 (7.4)	12.3 (1.65)	58.1 (15.2)	50.1 (2.08)
P = 0.268 (− 8.45 to 2.67)		P = 0.388 (− 9.98 to 4.21)		P = 0.123 (− 1.59 to 10.92)		P = 0.156 (− 3.75 to 19.75)	

## Discussion

This study evaluated the usability of a high-fidelity prototype GUI which was co-designed to enable PwS to choose from a range of exercise programmes and view real time feedback of their exercise performance during exercise. Two sequential versions of the GUI were evaluated with two user groups using online remote media with version 2 amended in response to usability problems detected on version 1 and extended to offer a range of programme choices. The use of a remote testing method to evaluate the usability of the new technology is reported which denotes a solution to the challenges associated with face-to-face usability evaluation with users of rehabilitation technologies. The value of different testing approaches is also reflected which will guide future research and design teams in the selection of tasks and analysis methods.

Integrated multiple methods of usability evaluation were implemented to detect usability problems and evaluate the user experience. Empirical, performance-based metrics including task completion rates and task duration were used to evaluate the usability of the GUI. In comparison, the ‘think aloud’ data and video footage captured qualitative insights into the users’ experience and facilitated identification of specific usability issues across all of the a-priori categories. Triangulation of different usability evaluation methods increases the chance of identifying usability issues and heuristic evaluation conducted by usability experts may further enhance methodological robustness [31]. However, examples from the literature indicate high similarity between the findings detected through heuristic evaluation and usability testing with representative end users [29].

The ‘think aloud’ data and usability observations were combined to create a descriptive list of categorised issues. The total number of recorded usability incidents on version 1 was 100 with 22 different usability issues identified. Eight of the 22 detected issues were prioritised according to severity and frequency and directly addressed in version 2. The total number of usability incidents on version 2 was 86, with 24 new usability issues identified. Most of these were associated with the new, extended programme menus, indicating that the amendments made to the ‘quick start’ menu did improve usability. This descriptive approach will enable specific usability issues to be ranked and addressed on future iterations of the interface [47]. Although the ‘think aloud’ data enabled insight into participant’s experience of navigating the GUI, comparable usability studies have captured rich qualitative data through focus groups or interviews to gain a more in-depth understanding of the participant’s perspectives on a novel technology [14, 21].

Although the amendments implemented on version 2 of the ‘quick start’ menu did improve its usability, the occurrence and seriousness of usability problems detected on version 2 suggests that further amendments are required before the technology is implemented. The ability to stop assisted movement quickly and call for assistance is a priority for safe use of power assisted exercise and the EU group were slower to complete this task on version 2 compared with version 1. On reviewing version 2 it was recognised that the ‘help’ icon was positioned more peripherally on the menu bar. This is particularly pertinent considering impairment in spatial awareness is widely reported amongst PwS which can impact ability to process visual input [43]. The use of red, centralised icons has therefore been recommended to ensure rapid activation of safety functions such as ‘stop’ or ‘quit’ on devices designed for PwS [26].

Task completion and task duration data benchmarked against the commercial target indicated that the ‘Quick Start’ programme on both versions of the GUI would enable users to commence exercise independently and within the required timescales. Comparison of task duration between version 1 and version 2 indicated a non-significant decrease in task duration amongst the PU participants and significant decrease for Task 1.1 amongst EU participants. This apparent improvement in usability may be attributed to the changes implemented on version 2. It is also possible that repeated exposure to the GUI may have contributed to the participant’s ability to navigate through it more quickly [48].

The safety and operational usability categories exemplified the divergence which can exist between operational efficiency and safety. Adjustments implemented on version 2 did reduce the occurrence of operational and safety problems, although access to support and supervision will need to be monitored during implementation of the technology. The co-designed GUI was intended to promote user independence, although the value of a supported induction to the equipment and availability of support throughout exercise was emphasised during the co-design stages of the research programme [13]. The safety of rehabilitation technologies is service and setting specific [32, 49]. Factors which should be considered in the implementation of rehabilitation devices in stroke rehabilitation include physical space, staff capacity, user ability and technological features [49].

One of the key features for the new technology was the introduction of effort detection capability and provision of biofeedback to enable users to observe, adjust and compare their exercise performance to previous sessions. Sophisticated gamification, augmented or virtual reality technology was beyond the resource available for this early iteration of the GUI but could be potentially incorporated in the future. The effort feedback dial featured on version 1 was widely misinterpreted as an indication of remaining duration; the dial was replaced by the effort balloon on version 2 which was very quickly understood by nearly all participants. Identification of the misinterpretation was detected through the ‘think aloud’ data. Analysis of think aloud data in the evaluation of digital apps for use by older adults has previously enabled categorisation of usability issues according to severity and types of barrier detected [48]. This exemplifies the value of ‘think aloud’ data compared with usability studies which have focussed on user satisfaction and adverse events to quantify usability [36].

The baseline assessment on version 2 was intended to create an individualised prescription for each user. Baseline assessment has been previously integrated with gaming technologies for PwS to develop a programme which was adaptive to different users and responsive to their fluctuating cognitive and motor ability [32, 48]. The purpose of the paler target intensity balloon introduced on version 2 was not clear to most participants and it was suggested that this would require verbal explanation to new users of the technology. Quantification of user performance was an area of dissonance between participants during the testing of version 1 and version 2. Positive reward about performance and a system which is responsive to all levels of ability is important to sustain user engagement [49]. Achievement of an effective and sustained exercise intensity is a challenge for providers of stroke recovery services as patients typically do not sustain the level of effort required for physiological benefit [7]. Assisted exercise with real-time feedback represents a potential solution as the motorised mechanism enables movement in the presence of motor impairment [50]. Sophisticated human-in-the-loop feedback systems synchronised with detected mechanical work rate have been piloted on similar technologies to optimise user attainment of target intensity [51].

The PSSUQ data captured an impression of the user experience and indicated that reported satisfaction was high with a non-significant increase recorded for version 2. However, the PSSUQ was not sensitive to specific usability issues and did not directly inform the amendments implemented on version 2. Comparable examples from the stroke literature have implemented modified user satisfaction questionnaires to evaluate and compare novel technologies [35]. Feingold-Polak et al. [35] reported higher user satisfaction for a robot guided exercise technology compared with a computer led system, although this difference was not statistically significant. User satisfaction scores were slightly higher amongst the EU group. Evaluation of similar assistive technologies has also reported higher satisfaction amongst expert users compared with professional users [14, 33]. It is possible that PU’s underestimate the ability of EU’s to navigate and operate digital interventions [33]. Service providers influence the extent to which assistive technologies are adopted and therefore addressing the viewpoints of PU representatives is important to ensure successful implementation [49].

The anticipated operators of digitised power assisted exercise equipment include leisure centres, community venues and rehabilitation services, with the target user groups comprising PwS, supported by therapy teams or exercise professionals. The use of remote testing methods enabled recruitment of participants who would have encountered practical barriers to attendance of face-to-face usability evaluation [39]. Rehabilitation and exercise professionals were recruited alongside PwS to capture the perspectives of multiple end users. This combination was intended to optimise detection of usability issues across the a-priori categories. PU participants detected more potential issues than the EU group during the testing of version 1. Interestingly, this disparity was not identified during the testing of version 2. It is possible that the EU participants required longer to understand the usability testing process and gain confidence in identifying and articulating potential issues. PU participants focussed on operational and safety issues, whilst the EU participants commented more on the programme effectiveness and user experience. Comparable usability studies examining stroke related technologies have selected only healthy participants to avoid the potential for bias associated with motor or cognitive impairment [32]. Expert users and those with lived experience remain under-represented in the development of new technologies and systems devised to optimise rehabilitation outcomes [26, 32]. Participants with neurological impairment have critical views on assistive technologies and their perspective should be complicit in the development and implementation of new equipment and products [33].

This study reported on stage three of a co-design and usability evaluation centred on the digital advancement of PAE equipment. Effort detection technology and a range of programme menus to guide the user through the setup process were developed and evaluated. The potential to further develop the technology was identified by research participants and the project team. Integration of heart rate sensors on the handles would enable specific monitoring of exercise intensity [52], whilst haptic or auditory signalling may improve accessibility of the technology for people with visual or perceptual impairments [53]. The real time feedback displayed on the GUI could be gamified or developed as an immersive virtual reality experience [54]. Development of a user identification system has been identified as a commercial priority and will enable data analytics, intelligent exercise prescription and connectivity with referring services [32].

This application of the medical device technology framework has integrated co-design techniques [13] with mixed method usability testing of two sequential versions of a new GUI. Due to the restrictions imposed by the COVID-19 pandemic, face to face usability testing was not possible and in order to navigate this challenge, synchronous remote testing was implemented. This study adds to the small number of examples of remote usability testing with hard to reach user groups which offers the advantage of cost effectiveness compared with in-house usability tests [55]. Although numerous usability issues were detected and addressed, the team recommend field testing of a late stage prototype prior to commercial implementation of the new technology. As the horizon for digital, robotic and assistive technologies expands, methodological approaches to optimise their design and usability are a priority in the field of rehabilitation engineering and robotics. The medical device technology framework ensures involvement of PU and EU groups and promotes a logical and yet iterative approach. The methods reported in this article have the potential to serve as an example in the development of future technologies.

## Study limitations

Data were collected during a period of national lockdown imposed by the government during the covid-19 pandemic; the original proposal to field test the GUI was adapted through implementation of remote media to enable virtual testing.

The objectives of the study were attained insofar as two sequential versions of the GUI were developed and evaluated capturing a diverse range of user experiences. The tasks which guided the usability testing were relevant to the proposed long-term use of the GUI and were effective in highlighting usability problems.

Several limitations are acknowledged in that the remote testing of a technology devised for venue-based exercise inevitably situated the user experience out of context. Although the sample size was calculated through application of the probabilistic model of problem discovery, this method was developed for non-clinical populations [40]. Given the complex cognitive, perceptual and motor impairments associated with stroke, a larger sample of EU participants would have reduced the likelihood of errors due to over or under representation. Measurement of the degree of motor or sensory impairment alongside cognitive and perceptual changes amongst the EU group was not conducted. The heterogenous nature of the sample means that results cannot be viewed as conclusive to the whole stroke population. On several occasions, participants commented that the usability problems encountered would have been less likely to occur if they had been engaged with the machine in a real-world setting such as in a gym environment or in a rehabilitation centre. However, the remote technology did enable more effective capture of the data. Stage 3 of the Medical Device Technology framework does stipulate real field testing of prototypes and this has been previously achieved by design teams who have conducted usability trials within the home environment [21]. In addition, field testing enables identification of technical problems due to hardware issues [35].

The testing procedure was dependent on reliable internet connectivity, access to a digital device and an ability to use Zoom software. This excluded individuals with limited digital access or ability from participation which is an area of increasing concern in healthcare provision and research [56]. Although the ‘think aloud’ data allowed some exploration of the participants’ qualitative perspective, the approach to data collection and analysis was primarily empirical. Comparable usability studies have included semi structured interviews to capture an in-depth insight into the users’ perspective and experience [33]. The same sample of participants tested version 1 and version 2 of the GUI which enabled direct intra-subject comparison between the versions. However, it is acknowledged that this may have introduced bias as the amendments were based on the participant’s initial feedback [31]. Introduction of new participants to version 2 would have strengthened the design of the study. The remote testing methods reported in this study have the potential to be applied to the evaluation of other user interfaces synched with rehabilitation technologies [39]. However, other widely reported barriers to adoption of rehabilitation technologies which include donning, doffing and set-up time require some face-to-face interaction between the participants and the research team.

## Conclusions

Robust co-design and usability evaluation methods are integral to the development and implementation of new assistive technologies in stroke rehabilitation. Remote testing of two sequential versions of a co-designed GUI with two user groups enabled identification of usability issues and evaluation of user satisfaction. The changes implemented on version 2 successfully addressed serious usability problems detected on version 1. However, the extended range of programme options introduced on version 2 created new usability problems; these mostly reflected concerns regarding therapeutic effectiveness of the technology rather than its operational efficiency or safety features. The ‘think aloud’ data combined with the observation of task walk performance was effective in detecting specific usability issues, whilst the task completion and duration data provided an indication of the operational readiness of the technology. The PSSUQ scores provided an overall impression of user satisfaction and enabled comparison between user groups and the two versions of the GUI.

The recruitment of EU and PU representatives enabled the research team to identify and address a range of usability problems. Diverse user perspectives were captured which improved the usability of the GUI and generated a vision for future technology advancement. The findings from this study will facilitate the transition from a high-fidelity prototype, to a market ready version of the technology which will enable end users of PAE to identify, monitor and progress rehabilitation goals. The next step in this process will comprise field testing of a late stage prototype in rehabilitation settings with a new sample of PU and EU representatives. The iterative model which underpins the medical device technology framework will ensure sustained user involvement throughout implementation and evaluation of the new technology.

## Supplementary Information

**Additional file 1.** Usability observation form.

**Additional file 2.** User by problem matrices.

## Data Availability

All datasets generated including usability observation forms and statistical calculations are available from the corresponding author on reasonable request.

## Abbreviations

EU: Expert user
GUI: Graphical user interface
PAE: Power assisted equipment
PSSUQ: Post Study System Usability Questionnaire
PU: Professional user
