# Supplementary material for: Evaluating the usability of a co-designed power assisted exercise graphical user interface for people with stroke
Source: J Neuroeng Rehabil. 2023 Jul 24;20:95. doi: 10.1186/s12984-023-01207-7 (PMC10364422; doi:10.1186/s12984-023-01207-7)
Supplement: Supplementary file 1 — Additional file 1. Usability observation form. [file 12984_2023_1207_MOESM1_ESM.docx]

**Additional file 1**

**Usability observation form**

**Task completion/duration record**

**
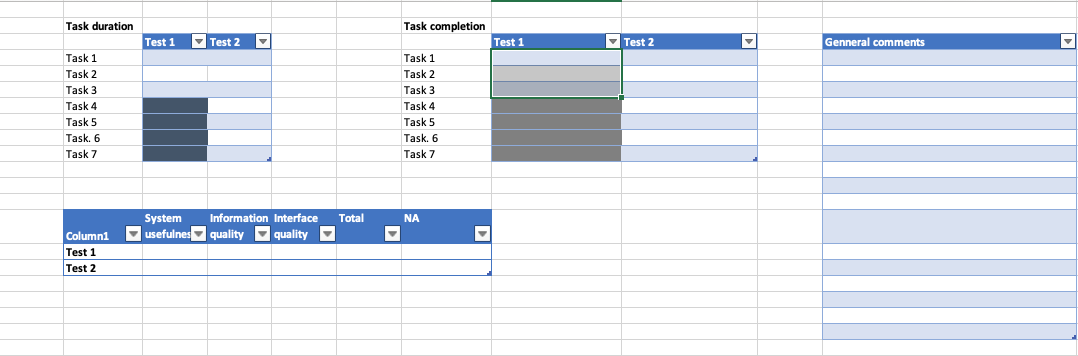
**

**Submenu example**

**
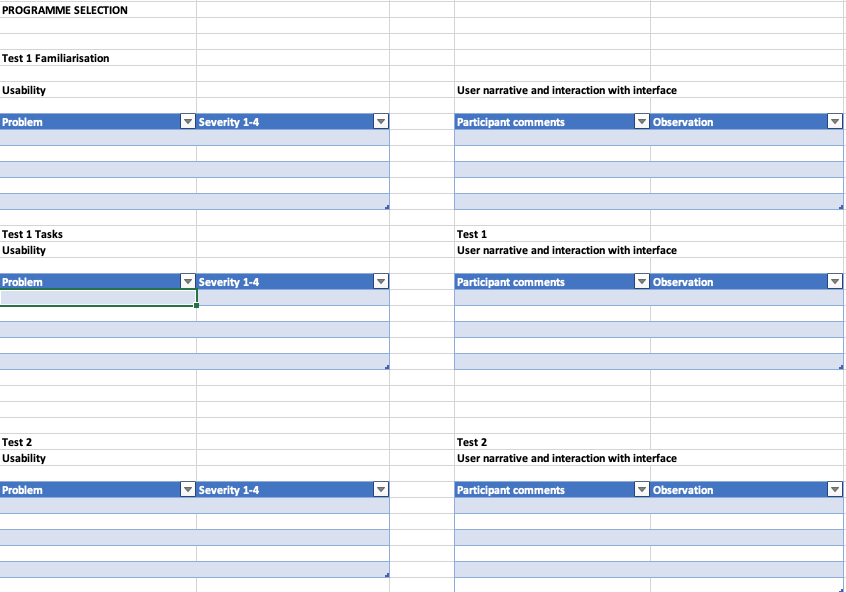
**
