# Supplementary material for: Evaluating the usability of a co-designed power assisted exercise graphical user interface for people with stroke
Source: J Neuroeng Rehabil. 2023 Jul 24;20:95. doi: 10.1186/s12984-023-01207-7 (PMC10364422; doi:10.1186/s12984-023-01207-7)
Supplement: Supplementary file 2 — Additional file 2. User by problem matrices. [file 12984_2023_1207_MOESM2_ESM.docx]

**Additional file 2**

**User by problem matrices**

**Table 1: Safety issues identified on v1**

|  | **EU1** | **EU2** | **EU3** | **EU4** | **EU6** | **EU7** | **EU8** | **EU9** | **EU 10** | **PU1** | **PU2** | **PU3** | **PU4** | **PU5** | **PU6** | **PU7** | **PU8** | **PU9** | **PU 10** | **Score** |
| --- | --- | --- | --- | --- | --- | --- | --- | --- | --- | --- | --- | --- | --- | --- | --- | --- | --- | --- | --- | --- |
| **S1.1** Help icon not visible enough |  |  |  |  |  |  |  |  | **4** |  |  |  | **4** |  | **4** | **4** |  |  | **4** | **S=4**  **F=5** |
| **S1.2** Difference between stop/pause/help unclear |  |  | **4** | **4** |  | **4** | **4** |  | **4** | **4** | **4** |  | **4** |  | **4** |  |  |  | **4** | **S=4**  **F=10** |
| **S1.3** ‘We’re on our way’ message/image missed | **3** |  |  |  |  |  | **3** |  |  |  |  |  | **3** |  | **3** |  | **3** |  |  | **S=3**  **F=5** |
| **S1.4** ‘Help’ function and purpose unclear |  |  |  |  |  |  |  |  |  | **3** |  |  | **3** |  | **3** |  |  |  |  | **S=3**  **F=3** |

**Table 2: Operational issues identified on v1**

|  | **EU1** | **EU2** | **EU3** | **EU4** | **EU6** | **EU7** | **EU8** | **EU9** | **EU 10** | **PU1** | **PU2** | **PU3** | **PU4** | **PU5** | **PU6** | **PU7** | **PU8** | **PU9** | **PU 10** | **Score** |
| --- | --- | --- | --- | --- | --- | --- | --- | --- | --- | --- | --- | --- | --- | --- | --- | --- | --- | --- | --- | --- |
| **O1.1** Repeated clicks to select intended duration |  |  |  |  |  |  |  |  |  |  |  |  |  |  |  | **3** |  |  |  | **S=3**  **F=1** |
| **01.2** Delay in identifying ‘play’ icon |  | **3** |  |  |  |  | **3** |  | **3** | **3** | **3** | **3** | **3** |  | **3** |  |  |  | **3** | **S=3**  **F=9** |
| **01.3** Repeated clicks to start exercise |  |  |  | **3** |  |  | **3** |  |  | **3** |  |  | **3** |  |  |  | **3** |  |  | **S=3**  **F=5** |
| **01.4** No option to resume workout after ‘help’ activation |  |  |  |  |  |  |  |  |  |  | **3** |  |  |  |  | **3** |  |  |  | **S=3**  **F=2** |
| **01.5** Error with duration selection | **3** |  |  |  |  |  |  | **3** |  |  |  |  |  |  |  |  |  |  |  | **S=3**  **F=2** |
| **01.6** Logout system unclear |  | **2** | **2** |  |  |  |  | **2** |  |  |  |  |  |  |  |  |  |  |  | **S=2**  **F=3** |
| **01.7** Curly icons for duration selection unclear |  |  |  |  |  |  |  | **2** |  |  | **2** |  |  |  | **2** |  |  |  | **2** | **S=2**  **F=4** |
| **01.8** The word ‘fob’ ambiguous |  |  |  |  |  |  |  |  |  |  |  |  | **1** | **1** |  |  |  |  |  | **S=1**  **F=2** |

**Table 3: Programme effectiveness issues identified on v1**

|  | **EU1** | **EU2** | **EU3** | **EU4** | **EU6** | **EU7** | **EU8** | **EU9** | **EU 10** | **PU1** | **PU2** | **PU3** | **PU4** | **PU5** | **PU6** | **PU7** | **PU8** | **PU9** | **PU 10** | **Score** |
| --- | --- | --- | --- | --- | --- | --- | --- | --- | --- | --- | --- | --- | --- | --- | --- | --- | --- | --- | --- | --- |
| **P1.1** No real time feedback on direction of effort |  |  |  |  |  |  |  |  | **2** |  |  |  |  |  |  |  |  |  |  | **S=2**  **F=1** |
| **P1.2** Body parts to be exercise not visualised |  |  |  |  |  |  |  |  |  |  |  |  |  |  |  |  |  |  | **2** | **S=2**  **F=1** |
| **P1.3** No option to input perceived effort |  |  |  |  |  |  |  |  |  |  |  |  | **2** |  |  |  | **2** |  |  | **S=2**  **F=2** |
| **P1.4** Real time effort feedback unclear | **3** | **3** |  |  | **3** |  | **3** |  | **3** | **3** | **3** | **3** | **3** | **3** | **3** | **3** | **3** |  |  | **S=3**  **F=13** |
| **P1.5** No real time feedback re symmetry |  |  |  |  |  | **3** |  | **3** |  |  |  |  |  |  |  | **3** |  | **3** |  | **S=3**  **F=4** |
| **P1.6** No real time feedback on distance covered |  |  |  |  | **2** |  |  |  |  |  |  |  |  |  |  |  |  |  |  | **S=2**  **F=1** |

**Table 4: User engagement issues identified on v1**

|  | **EU1** | **EU2** | **EU3** | **EU4** | **EU6** | **EU7** | **EU8** | **EU9** | **EU 10** | **PU1** | **PU2** | **PU3** | **PU4** | **PU5** | **PU6** | **PU7** | **PU8** | **PU9** | **PU 10** | **Score** |
| --- | --- | --- | --- | --- | --- | --- | --- | --- | --- | --- | --- | --- | --- | --- | --- | --- | --- | --- | --- | --- |
| **U1.1** Still image not inspiring |  |  |  |  |  |  |  |  |  |  | **1** |  |  |  |  | **1** |  |  |  | **S=1**  **F=2** |
| **U1.2** Col de Shapemaster not meaningful |  |  | **2** |  | **2** | **2** |  | **2** |  |  | **2** |  | **2** | **2** |  |  | **2** |  |  | **S=2**  **F=8** |
| **U1.3** Feedback metrics not meaningful |  | **2** |  |  |  |  | **2** |  | **2** | **2** |  |  | **2** | **2** | **2** |  | **2** |  | **2** | **S=2**  **F=9** |
| **U1.4** Missed ‘col-de-Shapemaster’ text |  |  |  | **2** | **2** |  | **2** | **2** |  |  | **2** |  |  |  | **2** |  |  |  | **2** | **S=2**  **F=7** |

**Table 5: Safety issues identified on v2**

|  | **EU1** | **EU2** | **EU3** | **EU4** | **EU6** | **EU7** | **EU8** | **EU9** | **EU 10** | **PU1** | **PU2** | **PU3** | **PU4** | **PU6** | **PU7** | **PU8** | **PU9** | **PU 10** | **Score** |
| --- | --- | --- | --- | --- | --- | --- | --- | --- | --- | --- | --- | --- | --- | --- | --- | --- | --- | --- | --- |
| **S2.1** Help icon not visible enough |  |  |  |  |  |  |  | **4** |  |  |  |  |  | **4** |  |  |  |  | **F=2**  **S=4** |
| **S2.2** Countdown to machine starting too short | **4** |  |  |  |  |  |  | **4** |  |  |  |  | **4** |  |  | **4** |  |  | **S=4**  **F=5** |
| **S2.3** Plus and minus icons could be misinterpreted for speed |  |  |  |  |  |  |  |  |  |  | **3** |  |  |  |  |  |  |  | **S=3**  **F=1** |
| **S2.4** Potential for user to proceed without assistance |  |  | **4** |  |  |  |  |  |  |  |  | **4** |  | **4** |  |  |  |  | **S=4**  **F=3** |

**Table 6: Operational issues identified on v2**

|  | **EU1** | **EU2** | **EU3** | **EU4** | **EU6** | **EU7** | **EU8** | **EU9** | **EU 10** | **PU1** | **PU2** | **PU3** | **PU4** | **PU6** | **PU7** | **PU8** | **PU9** | **PU 10** | **Score** |
| --- | --- | --- | --- | --- | --- | --- | --- | --- | --- | --- | --- | --- | --- | --- | --- | --- | --- | --- | --- |
| **02.1** Attempted to navigate to ‘hilly’ programme via quick start |  | **3** |  |  |  |  |  |  |  |  |  |  |  |  |  |  |  |  | **S=3**  **F=1** |
| **02.2** Concept of baseline assessment unclear |  |  | **3** | **3** |  |  | **3** |  |  |  | **3** |  | **3** |  |  | **3** |  | **3** | **S=3**  **F=7** |
| **02.3** Missed the ‘measurement/ programme’ subtext |  |  |  |  | **2** |  | **2** |  | **2** |  | **2** |  | **2** |  | **2** |  |  |  | **S=2**  **F=6** |
| **02.4** Assistance for baseline assessment unclear | **3** |  | **3** |  |  |  |  |  |  | **3** | **3** |  |  |  |  | **3** |  | **3** | **S=3**  **F=6** |
| **02.5** Cannot select duration in ‘my programme’ area |  |  | **3** |  |  |  |  |  |  | **3** |  | **3** |  |  | **3** | **3** |  |  | **S=3**  **F=5** |

**Table 7: Programme effectiveness issues identified on v2**

|  | **EU1** | **EU2** | **EU3** | **EU4** | **EU6** | **EU7** | **EU8** | **EU9** | **EU 10** | **PU1** | **PU2** | **PU3** | **PU4** | **PU6** | **PU7** | **PU8** | **PU9** | **PU 10** | **Score** |
| --- | --- | --- | --- | --- | --- | --- | --- | --- | --- | --- | --- | --- | --- | --- | --- | --- | --- | --- | --- |
| **P2.1** Real time effort feedback bubble not obvious |  |  |  |  |  |  |  | **3** |  |  |  |  |  |  |  |  |  |  | **S=3**  **F=1** |
| **P2.2** Purpose of target intensity circle not obvious | **3** |  | **3** |  |  | **3** | **3** | **3** |  | **3** |  | **3** | **3** | **3** | **3** | **3** |  | **3** | **S=3**  **F=12** |
| **P2.3** Missed the plus icon for intensity adjustment |  |  |  |  | **3** |  | **3** |  |  |  |  |  |  |  |  |  |  |  | **S=3**  **F=1** |
| **P2.4** Not clear that you can adjust intensity in real time |  |  |  |  |  |  | **3** |  | **3** |  |  |  |  |  |  |  |  |  | **S=3**  **F=2** |
| **P2.5** PwS would need longer to adjust effort |  |  |  |  |  |  |  | **3** |  |  |  |  |  |  |  |  |  |  | **S=3**  **F=1** |
| **P2.6** No temporal graphics on real time feedback |  |  |  |  |  |  |  |  |  | **2** |  | **2** |  |  |  | **2** |  |  | **S=2**  **F=3** |
| **P2.7** Speed adjustment option needed |  |  |  |  |  |  |  |  |  |  | **2** |  |  |  |  |  |  |  | **S=2** |
| **P2.8** Selected intensity not displayed |  |  | **2** |  |  |  |  | **2** | **2** | **2** |  |  |  |  | **2** | **2** |  | **2** | **S=2**  **F=7** |
| **P2.9** No feedback if you do click plus or minus icons | **2** |  | **2** |  |  |  |  | **2** |  | **2** |  |  |  |  |  |  |  |  | **S=2**  **F=4** |
| **P2.10** No real time feedback on heart rate |  |  |  |  |  | **2** |  |  |  |  |  |  |  |  | **2** |  |  |  | **S=2**  **F=2** |

**Table 8: User experience issues identified on v2**

|  | **EU1** | **EU2** | **EU3** | **EU4** | **EU6** | **EU7** | **EU8** | **EU9** | **EU10** | **PU1** | **PU2** | **PU3** | **PU4** | **PU6** | **PU7** | **PU8** | **PU9** | **PU 10** | **Score** |
| --- | --- | --- | --- | --- | --- | --- | --- | --- | --- | --- | --- | --- | --- | --- | --- | --- | --- | --- | --- |
| **U2.1** Unquantified metrics do not represent anything | **2** |  |  |  | **2** |  | **2** |  |  |  | **2** |  |  | **2** | **2** |  |  |  | **S=2**  **F=6** |
| **U2.2** Shapemaster island concept  inconsistent |  |  |  |  |  |  | **1** |  |  |  | **1** |  |  |  | **1** |  |  |  | **S=1**  **F=3** |
| **U2.3** No feedback on symmetry on results page |  |  |  |  |  |  |  |  |  |  |  |  |  |  | **2** |  | **2** |  | **S=2**  **F=2** |
| **U2.4** Need to include intensity level in results |  |  |  |  |  |  |  |  |  |  |  |  |  | **2** |  |  |  | **2** | **S=2**  **F=2** |
| **U2.5** Easy to miss Shapemaster Island text |  | **1** |  |  |  | **1** |  | **1** |  |  | **1** |  |  |  |  |  |  |  | **S=2**  **F=4** |
| **U2.6** Missed decimal point on 4.6km |  |  |  | **1** |  |  | **1** |  |  |  |  |  |  |  |  |  |  |  | **S=1**  **F=2** |
